# Supplementary material for: VOLTA: an enVironment-aware cOntrastive ceLl represenTation leArning for histopathology
Source: Nat Commun. 2024 May 10;15:3942. doi: 10.1038/s41467-024-48062-1 (PMC11087497; doi:10.1038/s41467-024-48062-1)
Supplement: Supplementary file 1 — Supplementary Information [file 41467_2024_48062_MOESM1_ESM.pdf]

Supplementary Information

Supplementary Note 1: Datasets

In this study, we used 6 datasets from 3 tissue types. The datasets included CoNSeP [21], NuCLS [22], PanNuke [40] (we refer to the breast and colon samples of this dataset as 2 different datasets), Lizard[41], and an internal dataset called Oracledataset. The details of each dataset can be found in Supplementary Table 1.

CoNSeP

This dataset consists of 41 H&E (scans of tissues stained with Hematoxylin and Eosin) tiles obtained from colorectal tissues. These tiles were extracted from a total of 16 whole slide images acquired from a single patient. Each tile is captured at a magnification scale of 40x and has dimensions of $1,000 \times 1,000$ pixels.

For each tile, two associated masks are provided: an instance mask that assigns a unique number to each individual cell, and a type mask that assigns a number corresponding to the cell's type. The dataset encompasses seven primary cell categories, including normal epithelial, malignant epithelial, inflammatory, endothelial, muscle, fibroblast, and miscellaneous cells.

It's worth noting that, in line with the original paper's recommendations, we have grouped normal and malignant epithelial cells together under the "epithelial" category. Similarly, the "spindle-shaped" category encompasses muscle, fibroblast, and endothelial cells. Therefore, the final dataset comprises four distinct cell groups: "epithelial", "spindle-shaped", "inflammatory", and "miscellaneous".

The train/test split for this dataset follows the same partitioning as the original dataset, as specified in the original paper.

NuCLS

This dataset comprises a total of 1,744 image tiles obtained from 124 breast cancer slides/patients sourced from the TCGA dataset, which was collated from 18 different institutions. The image tiles vary in size but are approximately 300×300 pixels in dimensions.

For each tile, two associated masks are provided: an instance mask that assigns a unique number to each individual cell, and a type mask that assigns a number corresponding to the cell's type. Within this dataset, there are originally 12 distinct cell types, including tumor, fibroblast, lymphocyte, plasma, macrophage, mitotic, vascular endothelium, myoepithelium, apoptotic body, neutrophil, ductal epithelium, and eosinophil cells. However, in alignment with the paper"s recommendations, these individual subtypes have been grouped into 5 overarching superclasses. These superclasses consist of "tumor" (comprising tumor and mitotic cells), "stromal" (comprising fibroblast, vascular

endothelium, and macrophage cells), "sTILs" (comprising lymphocyte and plasma cells), "apoptotic cells," and "others."
In our model evaluation, we designated the first fold of this dataset for testing purposes, while the remaining portion of the dataset was employed for training.

PanNuke
This dataset encompasses image tiles derived from 19 distinct tissue types, with breast and colon tissues being the most prevalent among them (number of patients are not disclosed in the original paper). The images were acquired using a 40x magnification scale, and the cell types featured in the dataset encompass neoplastic, epithelial, inflammatory, connective, and dead cells.
For each tile, two associated masks are provided: an instance mask that assigns a unique number to each individual cell, and a type mask that assigns a number corresponding to the cell's type. For the purposes of this study, our primary focus was on the breast and colon cells due to their larger representation compared to the other tissue types. While the colon portion of the dataset incorporates all five cell categories, the breast tissue is notable for the absence of dead cells, resulting in a total of four cell types specific to this tissue.
The dataset is organized by the original paper into three distinct folds, with the first fold earmarked for training purposes and the third fold designated for testing. We also used the same designated folds for training and validation purposes.

Lizard
This dataset comprises colon tissue H&E image tiles. Even though The number of patients or slides has not been disclosed by the authors, the images are obtained from six distinct sources spanning multiple geographic locations worldwide. The image tiles were scanned at a magnification scale of 20x. The dataset encompasses six primary cell categories, including epithelial, connective tissue, lymphocyte, plasma, neutrophil, and eosinophil cells. Similarly, for each tile, two associated masks are provided: an instance mask that assigns a unique number to each individual cell, and a type mask that assigns a number corresponding to the cell's type.
This has been split into 3 folds by the original paper. In our model evaluation, we allocated the first fold for training purposes, while the third fold was utilized for testing and validation, similar to the original paper's design.

Oracle This dataset is an internal collection of ovarian tissue samples from British Columbia, Canada. It comprises a total of 192 Tissue Microarray (TMA) cores collected from 102 patients, each stained with H&E. Additionally, for each TMA core, there are corresponding multi-color Immunohistochemistry (IHC) scans of an adjacent tissue slice. These IHC images capture various biomarkers, including CD3 (T-cell), CD94 (Natural Killer cell), FoxP3 (T-cell), CD79a (B-cell), CD8 (killer T-cell), CD68 (macrophages), CD16a (natural killer cell), and PanCK+ (epithelial cancer cells). These biomarkers serve as

maps to identify and locate different types of cells within the corresponding TMA core.
This dataset was also manually inspected by a pathologist to ensure that artifacts are minimized.
In our efforts to align the IHC images with their respective cores, it's important to note that, due to the circular shape of the images, only 11 of them were visually matched successfully. The cells within these matched images were designated as the test set for our analysis.

SarcCell
This dataset originates from an internal collection of soft tissue sarcoma samples, specifically epithelioid sarcoma cases and 7 corresponding slides from 7 patients. Whole tissue sections were obtained from formalin-fixed paraffin-embedded source blocks and underwent staining using multiplex immunohistochemistry (IHC) techniques. The IHC markers used included CD3 (for T-cell identification, 1:100, Leica Biosystems), CD20 (for B-cell identification, 1:200, Biocare), and CD208 (for mature dendritic cell identification, 1:50, Novus Biologicals). In addition to the IHC staining, adjacent sections were also stained using H&E. These stained sections were then scanned at 40X magnification and co-registered with their corresponding IHC image. Due to the registration issues, we had to exclude the CD208 marker, resulting in two markers of CD3 and CD20 remaining. This dataset was also manually inspected by a pathologist to ensure that artifacts are minimized.

MastCell
This dataset originates from an internal set of 2 mastocytosis cases of non-specific tissue type. The two cases were serially sectioned 4 times, and stained with H&E, CD117, Tryptase, and H&E. The first two sections (H&E, CD117) were registered, and the CD117 stain was used to label positive cells. We selected the CD117 as our 'labelling' stain due to it's higher specificity in comparison to the Tryptase stained slide. To evaluate the performance of the models, we used a hand-picked set of 128 cells from both positive and negative cohorts, while the rest were used for the training of the models.

MiDOG
The MiDOG dataset originates from the MIDOG 2022 Mitosis Domain Generalization Challenge (from MICCAI 2022) [42]. The dataset consists of 254 human cancer cases (breast cancer, neuroendocrine tumor, and melanoma), and 149 canine cancer cases (lung cancer, lymphoma, and cutaneous mast cell tumor. For each case, the pathologist selected a $2mm^2$ area and annotated any mitotic figures within the area. This resulted in 9501 mitotic figures and 11051 non-mitotic figures.

Supplementary Table 1 Datasets summary. The datasets are distributed across 4 tissue and 18 cell types to demonstrate the utility of the method.

Dataset	Cell Type Count	Tissue Type	Magnification Scale	Total Number of Cells	Cell Types	Number of Institutions
CoNSeP	4	Colon	40×	24,319	Spindle, epithelial, inflammatory, miscellaneous	1
NuCLS	5	Breast	20×	51,986	Tumor, stromal, sTIL, apoptotic, miscellaneous	18
PanNuke Breast	4	Breast	40×	34,806	Neoplastic, epithelial, inflammatory, and connective	> 2
PanNuke Colon	5	Colon	40×	23,831	Neoplastic, epithelial, inflammatory, connective, and dead	> 2
Lizard	6	Colon	20×	277,654	Epithelial, connective, lymphocyte, plasma, neutrophil, eosinophil	> 7
Oracle	3	Ovarian	40×	265,580	Tumor, T-cell, B-cell, and Natural Killer	1
SarcCell	2	Soft Tissue	40×	65,403	T-cell, B-cell, and dendritic	1
Mast Cell	2	Skin	40×	20,334	Mast Cell, miscellaneous	1
MiDOG	2	Lung, Lymphoma, Breast, Neuroendocrine	40×	17,081	Mitotic, miscellaneous	1

Supplementary Note 2: Ablation Study

To study the effects of each component of the framework, we also conducted multiple ablation studies. In this section, we limited the training duration to 200 epochs and reduced the batch size to 512, and the studies were performed only on the CoNSeP and NuCLS datasets to decrease the time and memory requirements.

Masking cells in the environment patch In this section, we studied the effects of the cell masking operation. In this regard, instead of masking all cells present in the environment patch, we only masked the target cell (the one that already exists in the associated single cell image fed into the *Cell Block*). The results (Supplementary Table 2) showed that the masking operation increased the general performance of the model, implying that reduced bias towards other cells' types and more focus on the non-cellular environment such as tissue structure was important for environment integration.

Supplementary Table 2 Masking cells in the environment patch ablation study

	CoNSeP			NuCLS		
	AMI	ARI	Purity	AMI	ARI	Purity
w/o Masking	18.15%	11%	57%	26.3%	26.2%	**70.2%**
w/ Masking	**21.3%**	**15.4%**	**59.4%**	**26.82%**	**28.7%**	69.35%

Multi-cropping We compared the effect of multi-cropping (global-local cropping augmentations) with the conventional local-local relation where both of the augmentation pipelines in the *Cell Block* contained the cropping operation. Surprisingly, although multi-cropping showed a significant improvement on NuCLS dataset, it reduced the performance of the model on CoNSeP (Supplementary Table 3). We hypothesized that this was due to the fact that the local augmentations obtained in this setting were not diverse enough for the model to be properly trained as the CoNSeP dataset had fewer data samples. Therefore, we increased the number of training epochs on the CoNSeP dataset and compared the effect of multi-cropping again. Our results (Supplementary Table 4) demonstrated the positive effect of multi-cropping on the performance when the model was trained for a longer time.

Supplementary Table 3 Multi-cropping ablation study

	CoNSeP			NuCLS		
	AMI	ARI	Purity	AMI	ARI	Purity
w/o Multi-crop	**22.7%**	**21.4%**	**60.1%**	23%	26.2%	69%
w/ Multi-crop	21.3%	15.4%	59.4%	**26.82%**	**28.7%**	**69.4%**

Supplementary Table 4 Ablation study of multi-cropping on CoNSeP with longer epochs

	CoNSeP		
	AMI	ARI	Purity
w/o Multi-crop	22.7%	**20.24%**	57.5%
w/ Multi-crop	**25.5%**	19.3%	**63.2%**

Memory Bank Supplementary Table 5 shows the results of the unsupervised clustering performance in the presence and absence of the negative sample memory bank. These results confirmed that the presence of the memory bank is critical to the performance of the model.
Comparing the results of this table with that of Table 1 (which was trained on 500 epochs with a batch size of 1024), we found that although the performance over the CoNSeP dataset dropped, the model produced almost similar results on the NuCLS dataset. We hypothesized that this must be related to the size of the dataset. Therefore, as the NuCLS dataset was 2× larger than the CoNSeP, the model could converge to a steady performance with even fewer training epochs.

Supplementary Table 5 Memory bank ablation study

	CoNSeP			NuCLS		
	AMI	ARI	Purity	AMI	ARI	Purity
w/o Memory Bank	20.1%	**15.6%**	57.8%	22.5%	24.4%	66.6%
w/ Memory Bank	**21.3%**	15.4%	**59.4%**	**26.82%**	**28.7%**	**69.35%**

Environment Patch Size To assess the impact of the environment patch-size on the performance of the model, we measured the performance of the model on the CoNSeP dataset with different environment sizes. Our experiments show that increasing the size of the environment can consequently increase the clustering performance, while too large environment patches can also reduce this performance to some extent (Supplementary Table 6).

Ensembling We also compared the impact of using the trained momentum encoder instead of the backbone in the testing phase, and as Supplementary Table 7 suggests, using the momentum encoder improved the performance of the model. This finding was expected since the momentum encoder equation forms a model similar to Polyak-Ruppert averaging [33], aggregating the the encoder network weights across all training epochs.

Supplementary Note 3: Evaluation Metrics

Supplementary Table 6 Effect of environment patch size on the cell clustering performance of the model evaluated on the CoNSeP dataset. Env 50, Env 100, Env 200, and Env 300 represent the experiments conducted by the environment patch size of 50, 100, 200, and 300 pixels, respectively.

Model	Metric	ENV 50	ENV 100	ENV 200	ENV 300
VOLTA	AMI	14.3%	17.1%	**25.5%**	23.7%
	ARI	6.0%	10.7%	**19.3%**	14.7%
	Purity	50.1%	51.7%	**63.5%**	60.0%

Supplementary Table 7 Ablation study of ensembling

	CoNSeP			NuCLS		
	AMI	ARI	Purity	AMI	ARI	Purity
w/o Ensembling	20.7%	13.5%	58%	24.4%	25.2%	69.3%
w/ Ensembling	**21.3%**	**15.4%**	**59.4%**	**26.82%**	**28.7%**	**69.4%**

The clustering performance was measured based on multiple metrics, including Adjusted Mutual Information (AMI) [61], Adjusted Random Index (ARI) [62], and Purity of the identified cell clusters by the model and ground truth labels. In particular, AMI captures the agreement between two sets of assignments using mutual information while it is adjusted to mitigate the effect of chance on the score. On the other hand, ARI is the chance-adjusted form of the rand index [63], which calculates the quality of the clustering based on the number of matching instance pairs. Also, Purity measures how the samples within each cluster are similar to each other. In other words, it demonstrates whether each cluster is a mixture of different classes or not.

Since the ground truth cell labels are known during the evaluation phase (as well as the total number of ground truth cell clusters), we used the same number of clusters as the target number of groups to divide the model predictions. These numbers can be found in Supplementary Table 1.

Supplementary Note 4: Dunn Index and Silhouette Score

We have also included the results of Silhouette and Dunn index of our cell clustering method to compare it with the baselines. However, since these metrics are point-based metrics, they provide a small score which does not work well for the purpose of comparison. However, due to the large scale of the data (thousands of cells), we expect misclustering of some cells. Such misclusterings would penalize the value of the Silhouette and Dunn Index to an extent that they cannot be used for a proper comparison of the methods. That said, we also included these results in Supplementary Table 8. More specifically, Dunn index calculates the minimum distance between the points from two clusters and the maximum distances between the points within each cluster. Given that some of the cells are inevitably categorized within a different cluster (even the

misclassification of a single cell can cause this issue), the minimum distance will be equal to 0, which results in the Dunn Index to be equal to 0.

Supplementary Note 5: Point-Wise UMAP Visualization

Figure 1 illustrates the point-wise UMAP visualization of VOLTA. As can be seen in this figure, most cells with the same type are grouped close to each other within each dataset.

Supplementary Note 6: Bsseline UMAP Visualization

To compare our results with the baselines qualitatively, we also demonstrate the UMAP visualization for some of the best-performing baselines. In particular, Supplementary Figures 2 to 4 illustrate the UMAP visualizations for the Manual Features, Morphological Features, and SimCLR baselines, respectively.

Supplementary Note 7: Supervised Fine-tuning

The results of supervised fine-tuning can be found in Supplementary Table 9.

Supplementary Note 8: Model Size Comparison

Despite the fact that our model is capable of outperforming the supervised models, we noticed that it has 70% fewer parameters compared to HoVer-Net. However, one might relate this matter to the fact that Hover-Net performs 2 tasks of cell detection and classification, simultaneously. To address this, we only included the parameters of the common encoder and the classification head of this model to have an impartial comparison (Supplementary Table 10).

Supplementary Note 9: Color Normalization

Since the CoNSeP dataset contains the images from one patient and they were scanned in one institution, we excluded it from this experiment.

Supplementary Table 8 Unsupervised clustering of cell representations across different methods and datasets in terms of Dunn Index and Silhouette Score.

Model	Metric	CoNSeP	NuCLS	PanNuke Breast	PanNuke Colon	Lizard	Oracle	SarcCell	MastCell	MiDOG
Pre-trained ImageNet	Dunn Index	0.0%	0.0%	0.0%	0.0%	-	0.0%	0.0%	0.0%	0.0%
	Silhouette	-1.2%	-11.6%	-5.3%	-2.6%	-	-0.2%	2.9%	0.0%	19.0%
Morphological	Dunn Index	0.0%	0.0%	0.0%	0.0%	0.0%	0.0%	0.0%	0.0%	0.0%
	Silhouette	-1.6%	-6.8%	-11.9%	-7.0%	-7.6%	0.7%	4.8%	-1.0%	0.0%
Manual Features	Dunn Index	0.0%	0.0%	0.0%	0.0%	0.0%	0.0%	0.0%	0.0%	0.0%
	Silhouette	-15.0%	-6.6%	-7.5%	-4.0%	-10.0%	-6.7%	1.2%	0.0%	13.1%
DCAE	Dunn Index	0.0%	0.0%	0.0%	0.0%	-	-	-	0.0%	0.0%
	Silhouette	0.8%	-5.7%	-1.4%	-1.0%	-	-	-	3.8%	5.0%
SimCLR	Dunn Index	0.0%	0.0%	0.0%	0.0%	-	0.0%	0.0%	0.0%	0.0%
	Silhouette	2.5%	-2.1%	-0.6%	-1.9%	-	2.2%	5.1%	1.4	14.2
DINO	Dunn Index	0.0%	0.0%	0.0%	0.0%	-	-	0.0%	0.0%	0.0%
	Silhouette	-2.0%	-10.0%	-3.4%	-4.0%	-	-	2.5%	2.7%	12.1%
Volta (w/o Env)	Dunn Index	0.0%	0.0%	0.0%	0.0%	-	0.0%	0.0%	0.0	0.0
	Silhouette	2.6%	-3.3%	-5.0%	-6.3%	-	0.2%	3.7%	21.2%	23.0%
VOLTA	Dunn Index	0.0%	0.0%	0.0%	0.0%	0.0%	0.0%	0.0%	0.0%	0.0%
	Silhouette	4.0%	-3.3%	-3.2%	-1.8%	2.1%	3.3%	2.7%	11.2%	12.1%

Supplementary Table 9 Fine-tuning accuracy for CoNSeP and NuCLS datasets. The supervised baselines demonstrate the performance of the HoVer-Net and NuCLS models on the CoNSeP and NuCLS datasets, respectively.

	CoNSeP	NuCLS
Supervised Baseline	79.6%	77.5%
1-Layer MLP with frozen backbone	79.3%	75.7%
1-Layer MLP with unfrozen backbone	79.6%	75.8%
2-Layer MLP with frozen backbone	76.8%	74.5%
2-Layer MLP with unfrozen backbone	**80.22%**	76.3%

Supplementary Table 10 Model size comparison

	Inference Parameter Count
HoVer-Net	35.3M (common encoder: 25.6M, head: 9.7M)
Mask-RCNN in NuCLS	130M
Ours (1-Layer MLP)	**11.17M**
Ours (2-Layer MLP)	**11.43M**

Supplementary Table 11 Color normalization effect on unsupervised cell clustering

	w/o Color normalization			w/ Color normalization		
	AMI	ARI	Purity	AMI	ARI	Purity
VOLTA	26.1%	**25.6%**	70.3%	**26.8**%	22.9%	**70.8%**

Supplementary Table 12 Fine-tuning of NuCLS with color normalization

	NuCLS
Fully-Supervised baseline	77.5%
1-2 1-Layer MLP with unfrozen backbone	78.1%
2-Layer MLP with unfrozen backbone	**78.2%**

Supplementary Note 10: Cancer Subtype Clustering

In this section, we elaborate on the cancer subtype clustering pipeline and show the respective results of this process.

Cancer Subtype Clustering using VOLTA

To predict the cancer subtypes of a cohort of images, each image was divided into 512×512 patches, and cells were divided into multiple clusters using the K-means algorithm. For the ovarian cohort, we built a graph from the cells by connecting each cell to its 7 closest cells. Then, for each image, we measured the number cell type-to-cell type connections (.e.g., cell type 1-to-cell type 1, cell type 1-to-cell type 2) as well as the total number cells for each cell cluster. This resulted in a 110 dimensional vector (100 for cell-to-cell connections and 10 for cell type counts). Finally, we reduced the dimensionality of this vector to 10 using the Principal Component Analysis (PCA) and performed clustering to predict the cancer subtype. The final grouping process was conducted by using a hierarchical clustering algorithm with Ward's linkage method. For the endometrial cohort, we realized the the cell count captures enough information for the subtype clustering. Therefore, we omitted the graph construction stage and directly calculated the mean and standard deviation of the cell cluster counts across patches of each image. The extracted statistics were used to perform a hierarchical clustering of cases using the ward linkage method. The workflow of this pipeline can be found in Supplementary Figure 5.

Cancer Subtype Clustering using Patch-Based SSL

To compare the results of our model, we utilized the unsupervised patch representation learning proposed by [35]. Specifically, we used the pre-trained SSL ResNet18 model from [35] and treated it as a comparison baseline for the ovarian histotype and endometrial molecular subtype clustering. Specifically, we extracted features from the model for 224×224 patches and averaged these representations for the patches from the same core, and finally applied a hierarchical algorithm on top of these representations. The results of these experiments can be found in Supplementary Figure 14 for both datasets and both methods. Additionally, our quantitative results shown in Supplementary Table 14 demonstrate the superiority of our model objectively.

Cell Cluster Visualization

Supplementary Figure 12 depicts the boxplot of the cell area for each cell clusters of the ovarian dataset, and Supplementary Figure 13 shows the proportion of tumor 2, 4, and 5 clusters with respect to all the tumor clusters.

Supplementary Table 13 Distribution of cell clusters across epithelial ovarian cancer histotypes (with standard deviation - 12 cases).

	Case Count	Lymphocytes/Small Stromal	Tumor	Cells with Dark Nuclei	Stromal	Lymphocytes	Spindle-shape	Tumor/Stromal
Clear Cell	3	3.0 ±0.4%	59.5 ± 1.0%	1.6 ± 0.3%	8.8 ± 0.6%	0.4 ± 0.1%	12.5 ± 0.8%	14 ± 0.8%
Endometrioid	3	3.3 ±0.3%	57.2 ± 0.9%	0.4 ± 0.1%	10.6 ± 0.6%	0.3 ± 0.1%	0.3 ± 0.6%	18.4 ± 0.8%
High grade	3	3.6 ±0.3%	57.2 ± 0.9%	1.9 ± 0.2%	9.9 ± 0.6%	0.5 ± 0.1%	7.0 ± 0.5%	19.6 ± 0.8%
Low grade	3	8.0 ±0.4%	63.8 ± 0.7%	3.5 ± 0.3%	11.9 ± 0.5%	1.6 ± 0.1%	9.7 ± 0.4%	1.0 ± 0.2%

Supplementary Table 14 Comparison of subtype classification for Volta and Patch-based SimCLR on ovarian dataset 2 and endometrial dataset 2.

**Dataset**	**AMI**	**ARI**	**Purity**
	**Ovarian Datasets**		
VOLTA (ours)	**61.96%**	**72.09%**	**94.09%**
Patch-Based SimCLR	22.53	14.68	80.65
	**Endometrial Datasets**		
VOLTA (ours)	**4.50%**	**5.54%**	**61.93%**
Patch-Based SimCLR	0.99	1.66	56.76

Supplementary Table 15 Distribution of cell clusters across endometrial cancer molecular subtypes (with standard deviation - 19 patients). The non-MMRd group encompasses p53abn and POLE tumors.

	Case Count	Stromal and Inflammatory	Lymphocytes	Elongated Cells	Tumor	Inflammatory and Small Stromal	Medium-Sized Cells
MMRd	5	$6.8 \pm 0.03\%$	$9.6 \pm 0.04\%$	$10.3 \pm 0.04\%$	$54.3 \pm 0.06\%$	$0.4 \pm 0.008\%$	$18.6 \pm 0.05\%$
non-MMRd	14	$6.6 \pm 0.03\%$	$7.9 \pm 0.03\%$	$13.5 \pm 0.04\%$	$51.0 \pm 0.06\%$	$0.4 \pm 0.007\%$	$20.6 \pm 0.04\%$
P53abn	5	$6.3 \pm 0.2\%$	$7.3 \pm 0.03\%$	$12.9 \pm 0.04\%$	$53.9 \pm 0.05\%$	$0.4 \pm 0.007\%$	$19 \pm 0.04\%$
POLE	9	$6.8 \pm 0.03\%$	$8.2 \pm 0.03\%$	$13.8 \pm 0.04\%$	$49.1 \pm 0.05\%$	$0.3 \pm 0.007\%$	$21.56 \pm 0.04\%$

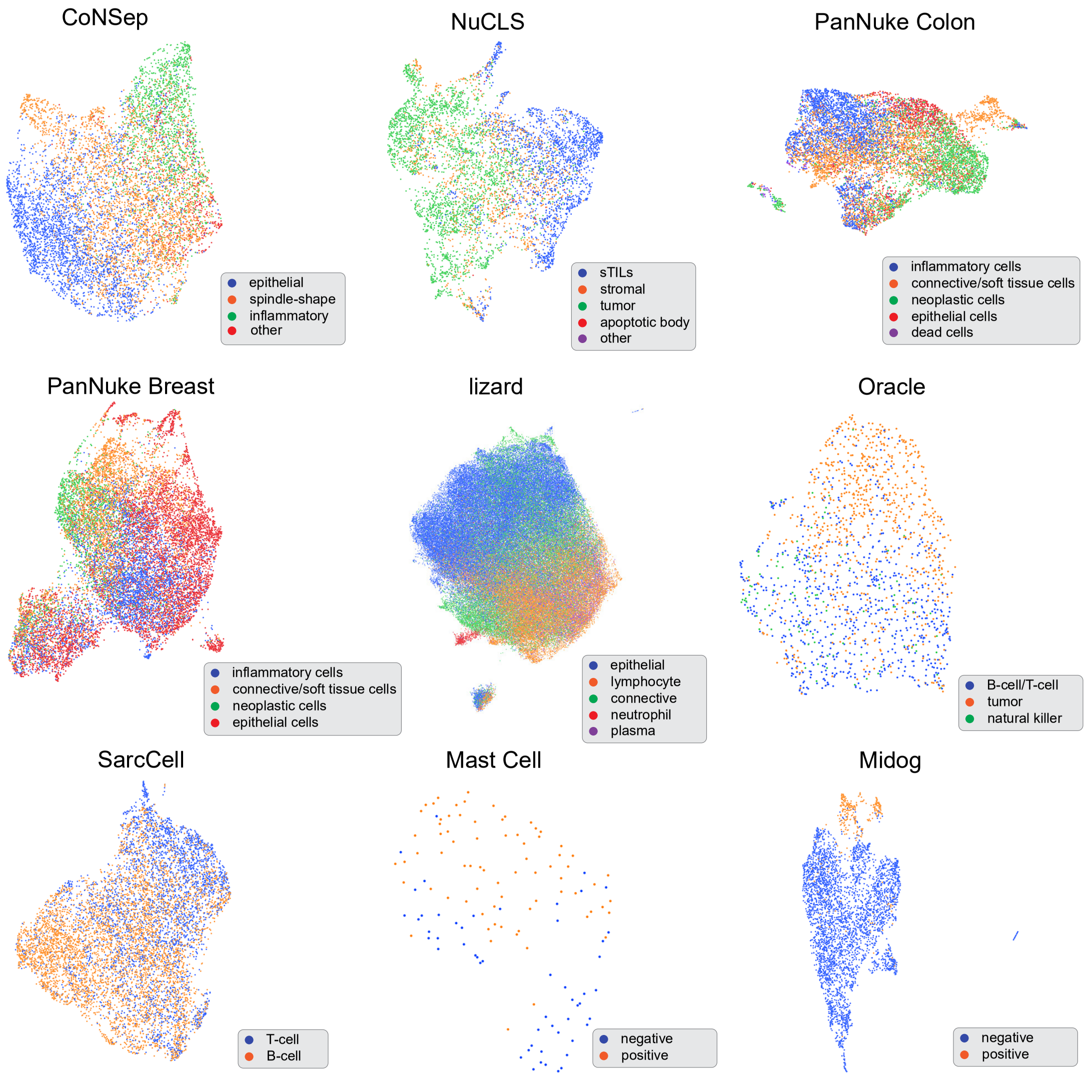

Supplementary Figure 1 Point-wise UMAP visualization. The point-wise UMAP visualization of VOLTA for different dataset (Source data are provided as a Source Data file).

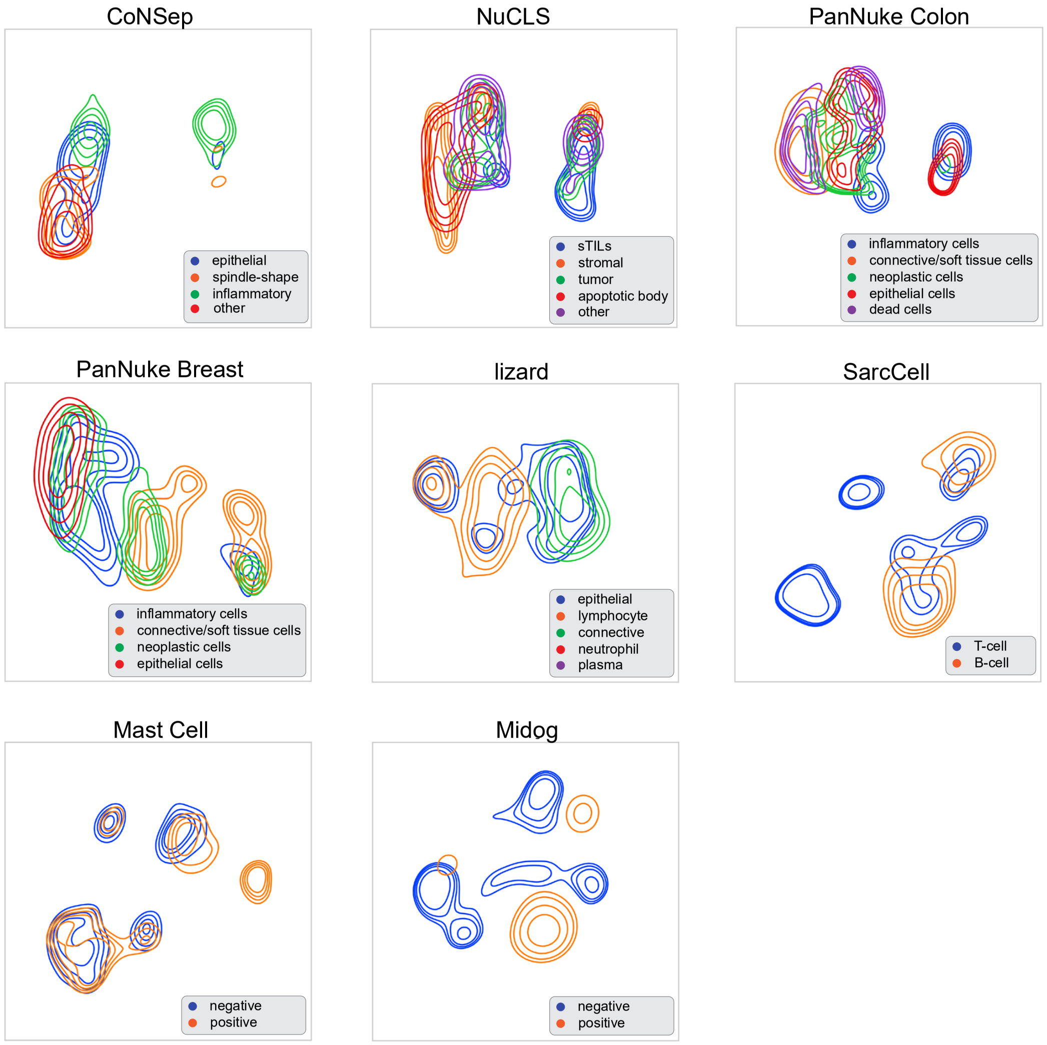

Supplementary Figure 2 Contour UMAP Visualization of Manual Features. The UMAP visualization of Manual Features baseline model for different dataset (Source data are provided as a Source Data file).

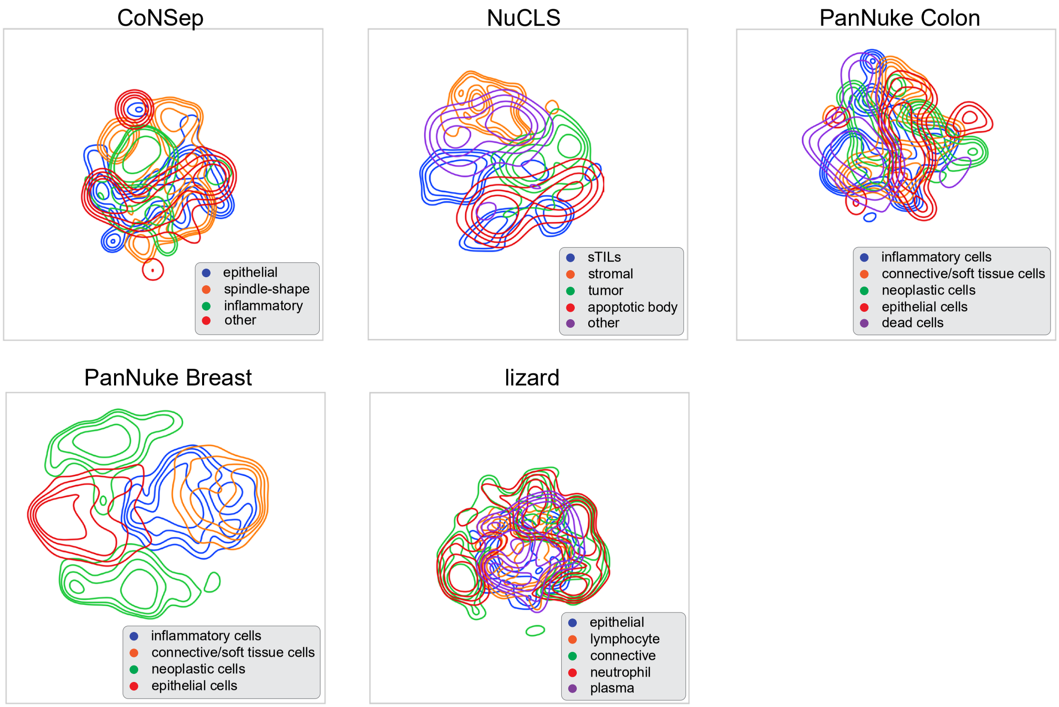

Supplementary Figure 3 Contour UMAP Visualization of Morphological Features. The UMAP visualization of Morphological Features baseline model for different dataset (Source data are provided as a Source Data file).

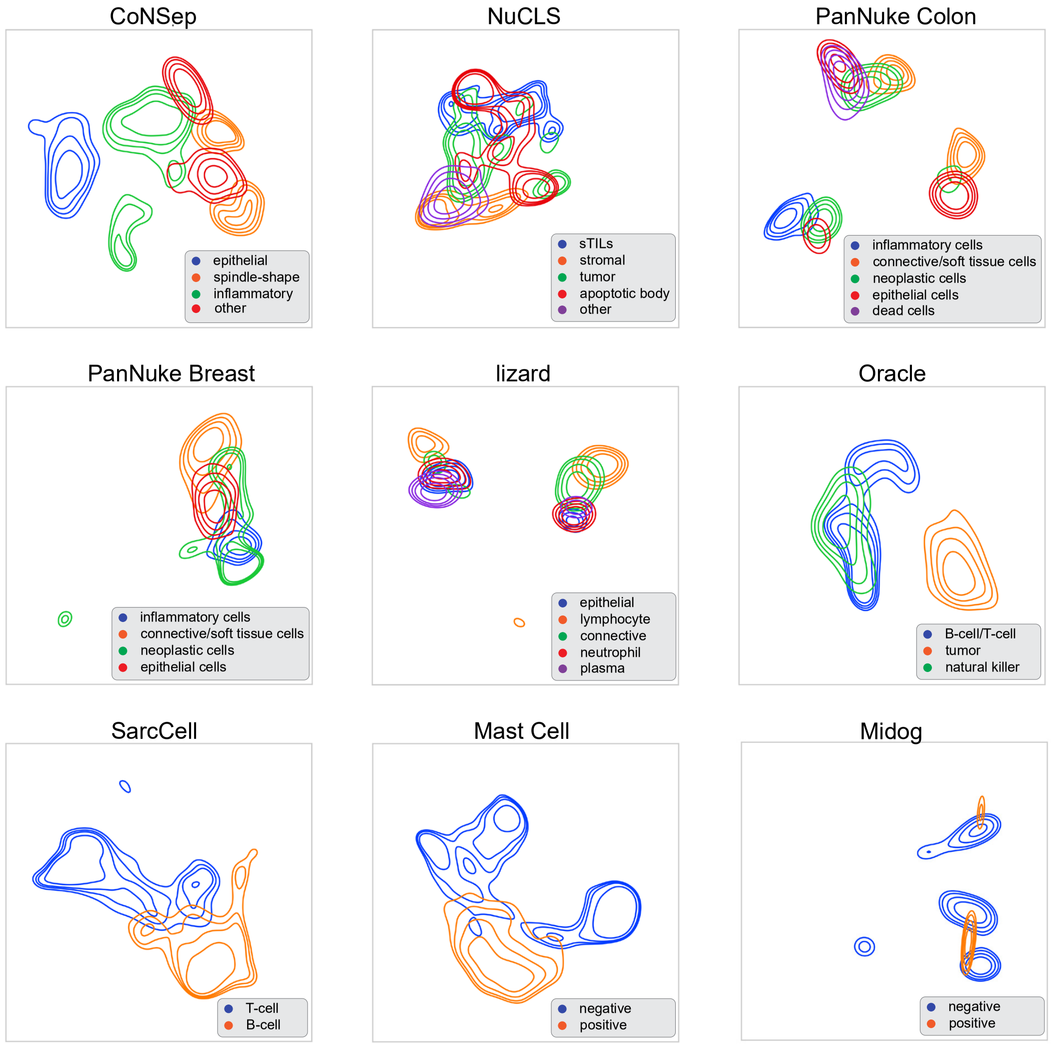

Supplementary Figure 4 Contour UMAP Visualization of SimCLR. The UMAP visualization of SimCLR baseline model for different dataset (Source data are provided as a Source Data file).

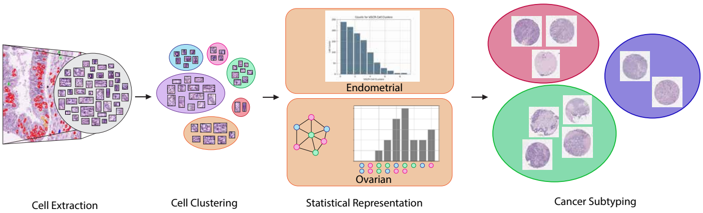

Supplementary Figure 5 Cancer Subtyping Workflow. Workflow of unsupervised cancer subtype classification based on the unsupervised cell representation learning (Source data are provided as a Source Data file).

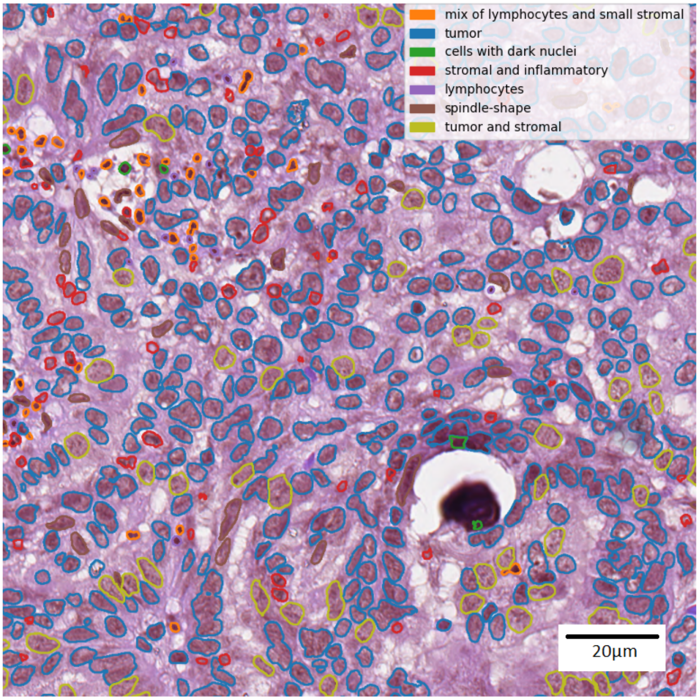

Supplementary Figure 6 Cell Cluster Visualization of Low-Grade Ovarian Cancer. Inferred cell cluster labels in a representative example of a low-grade serous ovarian carcinoma with psammomatous calcification. Cell cluster labels are depicted as coloured nuclear outlines, and represent morphologically-distinct clusters that are associated with cell types. Green captures cells with darkly staining nuclei, and brown labels spindled cells that are mostly stromal. The scale bar shows the scale relationship between the pixels and the actual metrical size (100 pixels equal to 20 micron). (Source data are provided as a Source Data file).

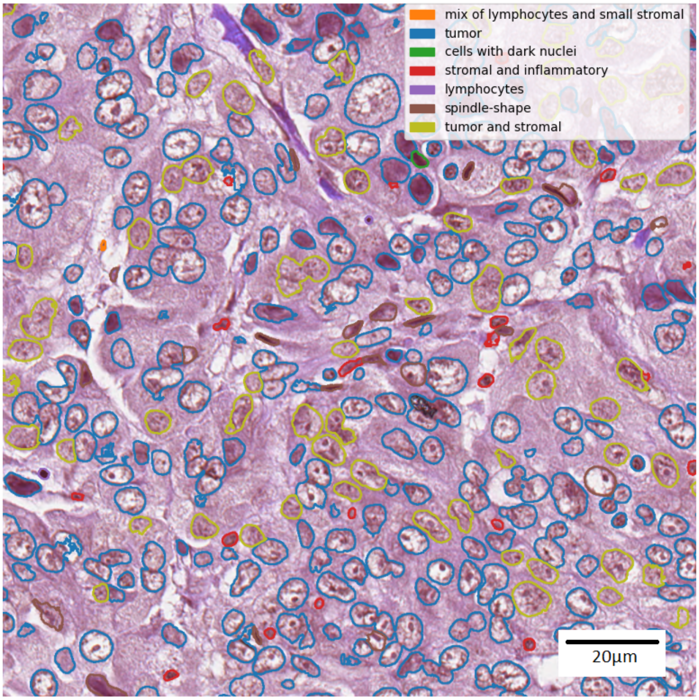

Supplementary Figure 7 Cell Cluster Visualization of High-Grade Ovarian Cancer. Inferred cell cluster labels in a representative example of a high-grade serous ovarian carcinoma. Cell cluster labels are depicted as coloured nuclear outlines, and represent morphologically-distinct clusters that are associated with cell types. Green captures cells with darkly staining nuclei, and brown labels spindled cells that are mostly stromal. The scale bar shows the scale relationship between the pixels and the actual metrical size (100 pixels equal to 20 micron). (Source data are provided as a Source Data file).

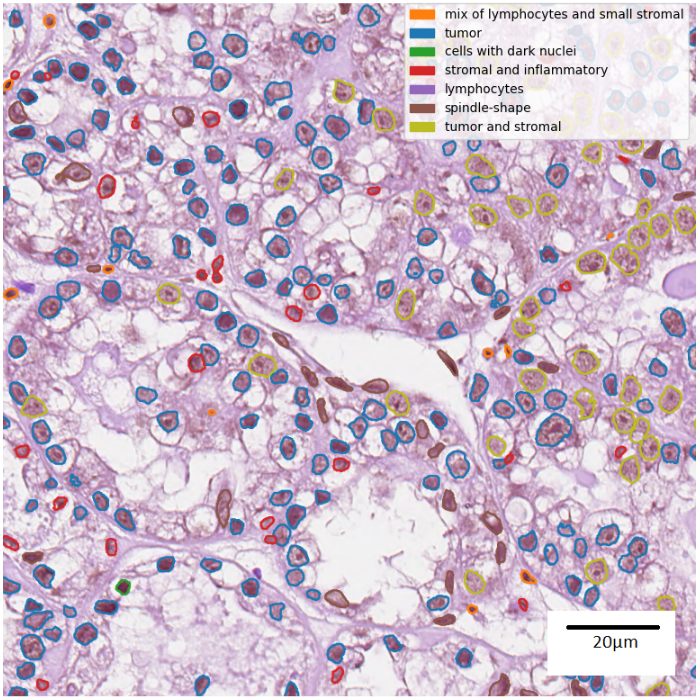

Supplementary Figure 8 Cell Cluster Visualization of Clear-Cell Ovarian Cancer. Ovarian - clear cell overlay visualization. The scale bar shows the scale relationship between the pixels and the actual metrical size (100 pixels equal to 20 micron). (Source data are provided as a Source Data file).

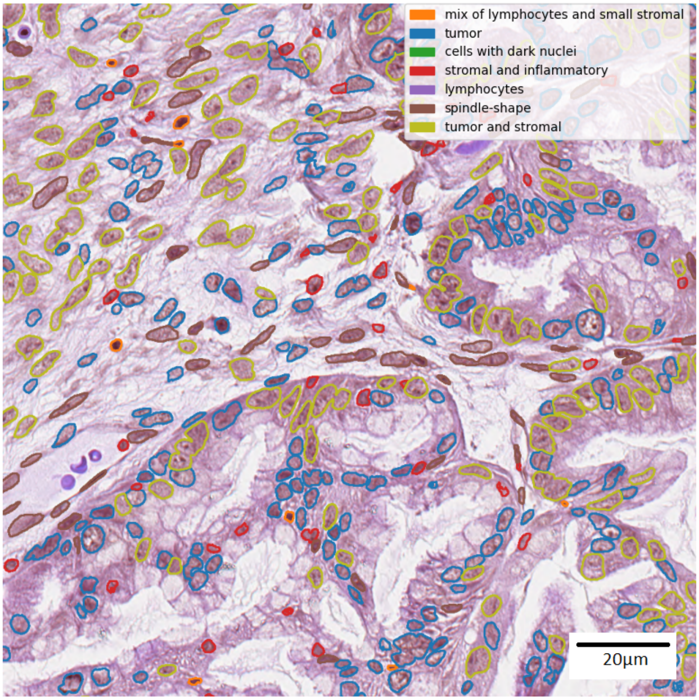

Supplementary Figure 9 Cell Cluster Visualization of Mucinous Ovarian Cancer. Inferred cell cluster labels in a representative example of a mucinous ovarian carcinoma. Cell cluster labels are depicted as coloured nuclear outlines, and represent morphologically-distinct clusters that are associated with cell types. Green captures cells with darkly staining nuclei, and brown labels spindled cells that are mostly stromal. In this example, blue also captures reactive stromal cells with plump nuclei. The scale bar shows the scale relationship between the pixels and the actual metrical size (100 pixels equal to 20 micron). (Source data are provided as a Source Data file).

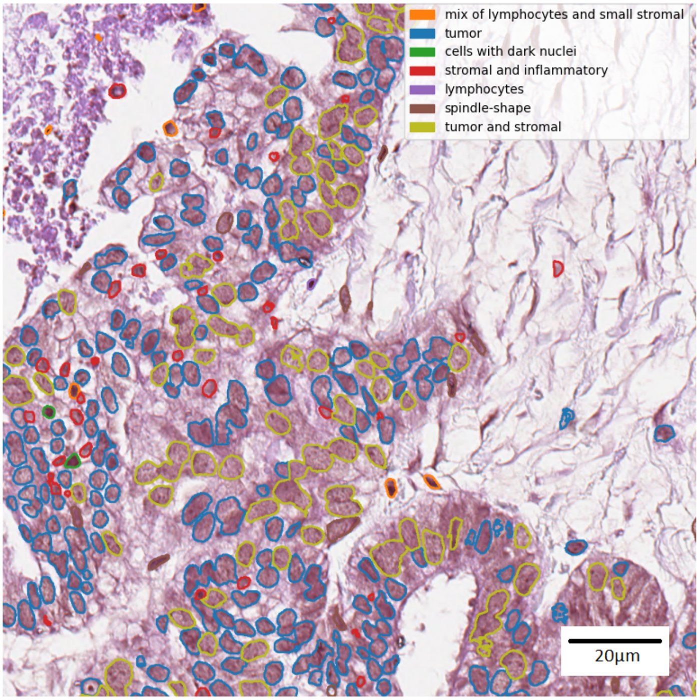

Supplementary Figure 10 Inferred cell cluster labels in a representative example of an endometrioid ovarian carcinoma. Cell cluster labels are depicted as coloured nuclear outlines, and represent morphologically-distinct clusters that are associated with cell types. Green captures cells with darkly staining nuclei, and brown labels spindled cells that are mostly stromal (Source data are provided as a Source Data file).

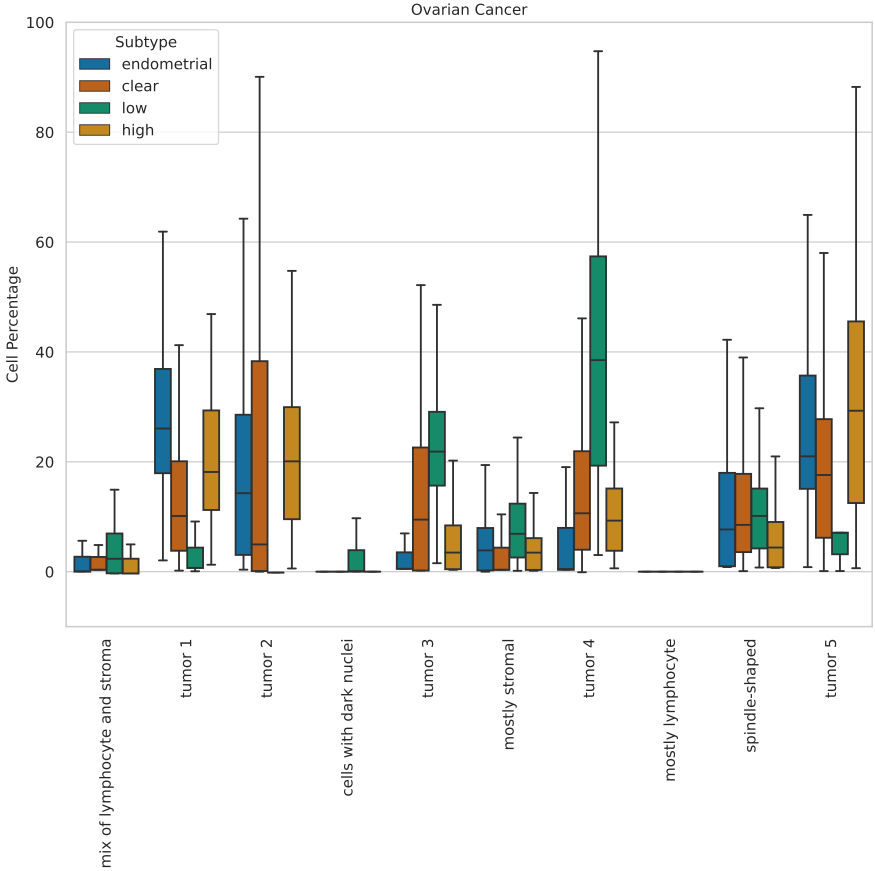

Supplementary Figure 11 Ovarian Cluster Count Distribution. Boxplot of cell cluster distribution across patches of the ovarian cancer dataset for all of the patients. The blue, orange, green, and red boxes represent the percentage of each cell cluster for endometrioid, clear-cell, low-grade serous, and high-grade serous histotypes of ovarian cancer (Source data are provided as a Source Data file).

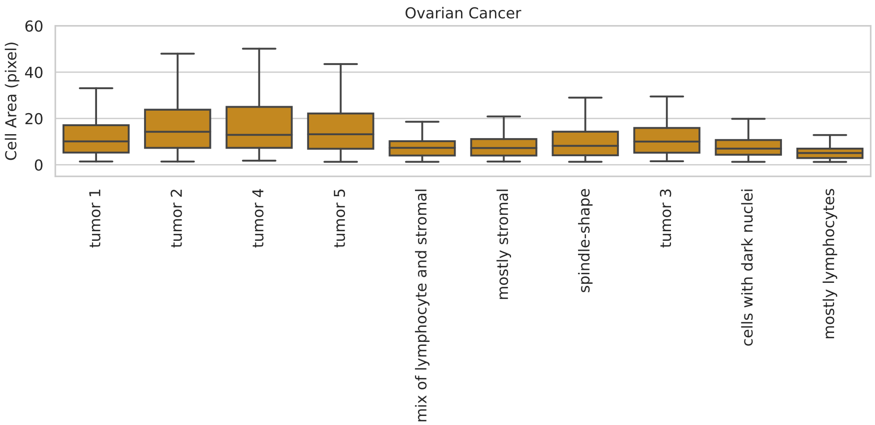

Supplementary Figure 12 Cell Area across Ovarian patients Cell area boxplot for cell clusters of the ovarian dataset (Source data are provided as a Source Data file).

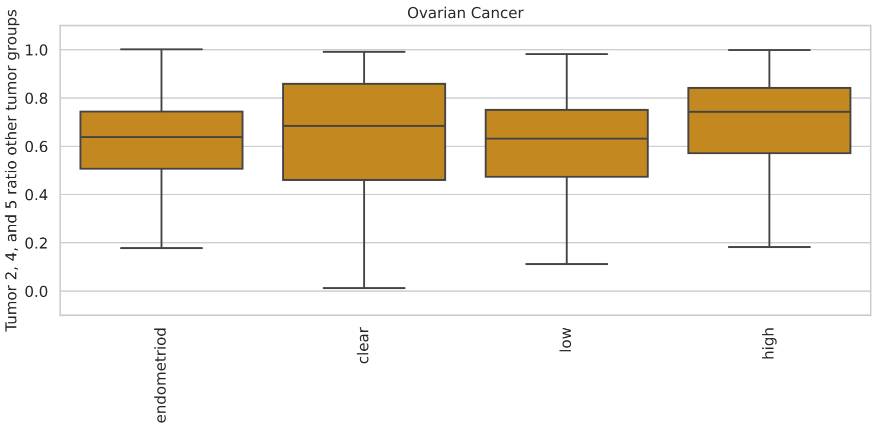

Supplementary Figure 13 Tumor Subcluster Ratio of Ovarian Cancer. Tumor 2, 4, and 5 clusters proportion ratio with respect to all the tumor clusters for each histotype (Source data are provided as a Source Data file).

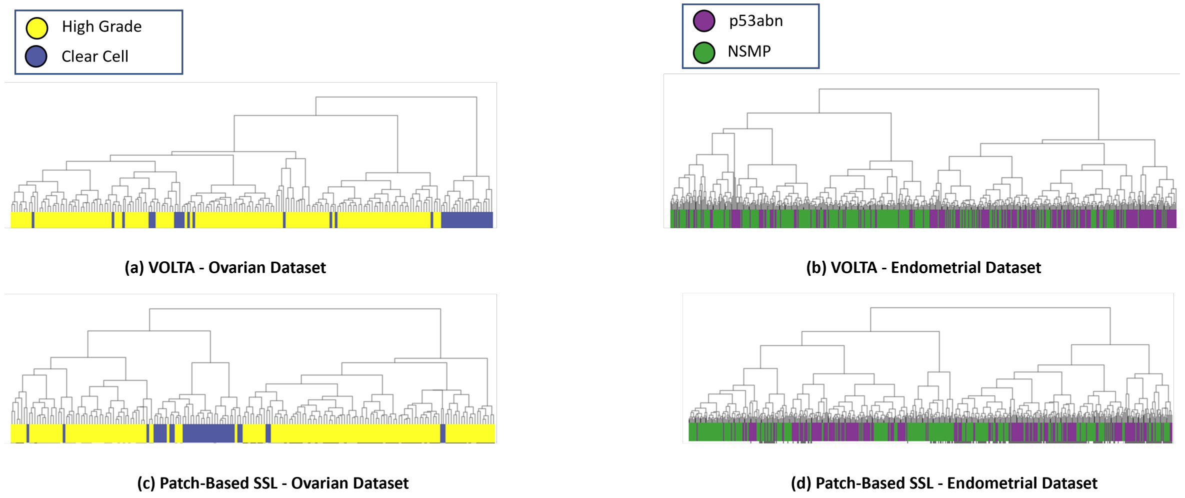

Supplementary Figure 14 Cancer Subtype Clustering Comparison. Qualitative comparison of cancer subtype clustering between VOLTA and patch-based SSL [35] on ovarian dataset 2 and endometrial dataset 2 (Source data are provided as a Source Data file).

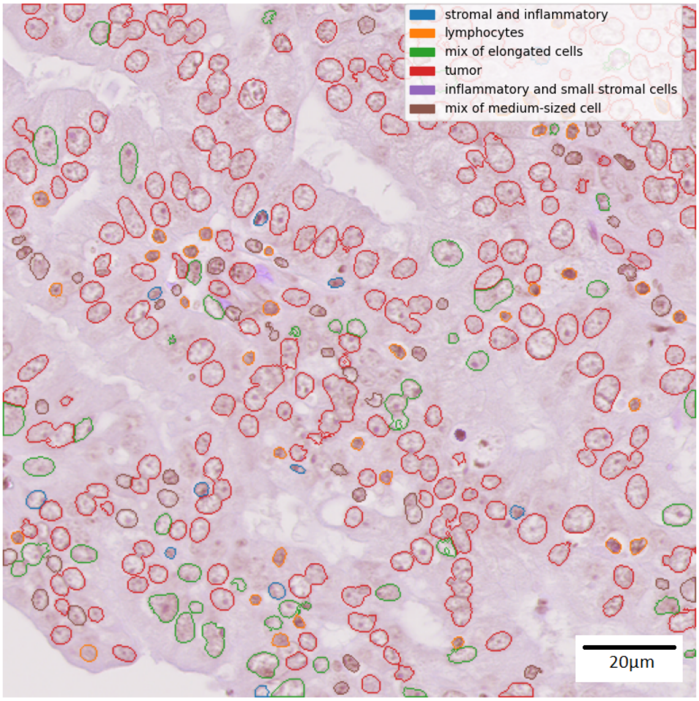

Supplementary Figure 15 Cell Cluster Visualization of POLE Endometrial Cancer. Inferred cell cluster labels in a representative example of a POLE-mutated endometrial carcinoma. Cell cluster labels are depicted as colored nuclear outlines, and represent morphologically-distinct clusters that are associated with cell types. Green cells comprise a mixture of stromal and cancer cells with elongated nuclei. Brown denotes cells with intermediate-sized nuclei, comprising a mixture of tumor, inflammatory, and stromal cells. In this example, some cancer cells are labeled green, highlighting the uncertainty of cell type classification in morphologically pleomorphic POLE tumors. The scale bar shows the scale relationship between the pixels and the actual metrical size (100 pixels equal to 20 micron). (Source data are provided as a Source Data file).

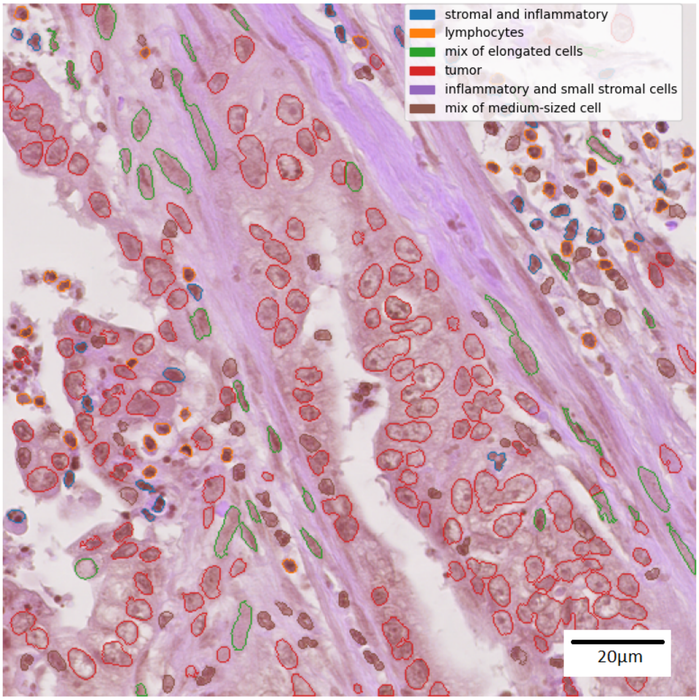

Supplementary Figure 16 Cell Cluster Visualization of MMRd Endometrial Cancer. Endometrial - MMRd overlay visualization. The scale bar shows the scale relationship between the pixels and the actual metrical size (100 pixels equal to 20 micron). (Source data are provided as a Source Data file).

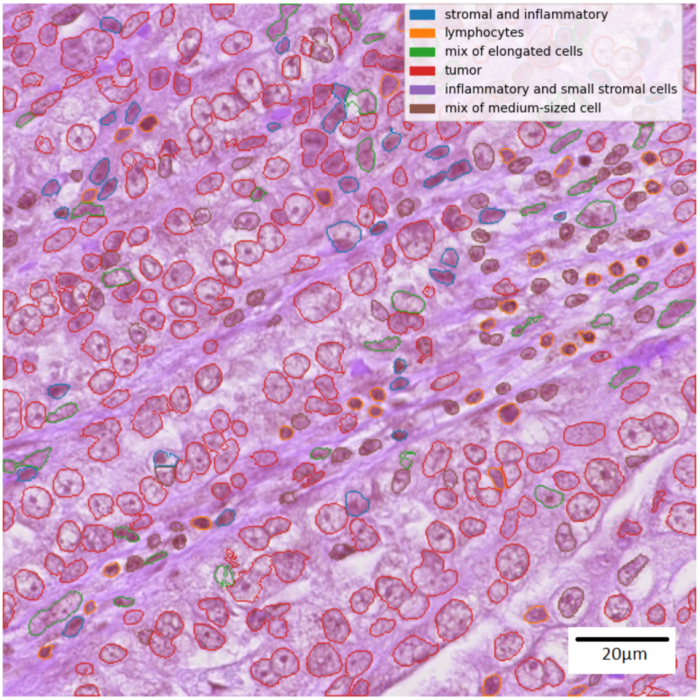

Supplementary Figure 17 Cell Cluster Visualization of p53-mutated Endometrial Cancer. Inferred cell cluster labels in a representative example of a p53-mutated endometrial carcinoma. Cell cluster labels are depicted as coloured nuclear outlines, and represent morphologically-distinct clusters that are associated with cell types. Green cells comprise mixture of stromal and tumour cells with elongated nuclei. Brown denotes cells with intermediate sized nuclei, comprising a mixture of tumour, inflammatory, and stromal cells. The scale bar shows the scale relationship between the pixels and the actual metrical size (100 pixels equal to 20 micron). (Source data are provided as a Source Data file).

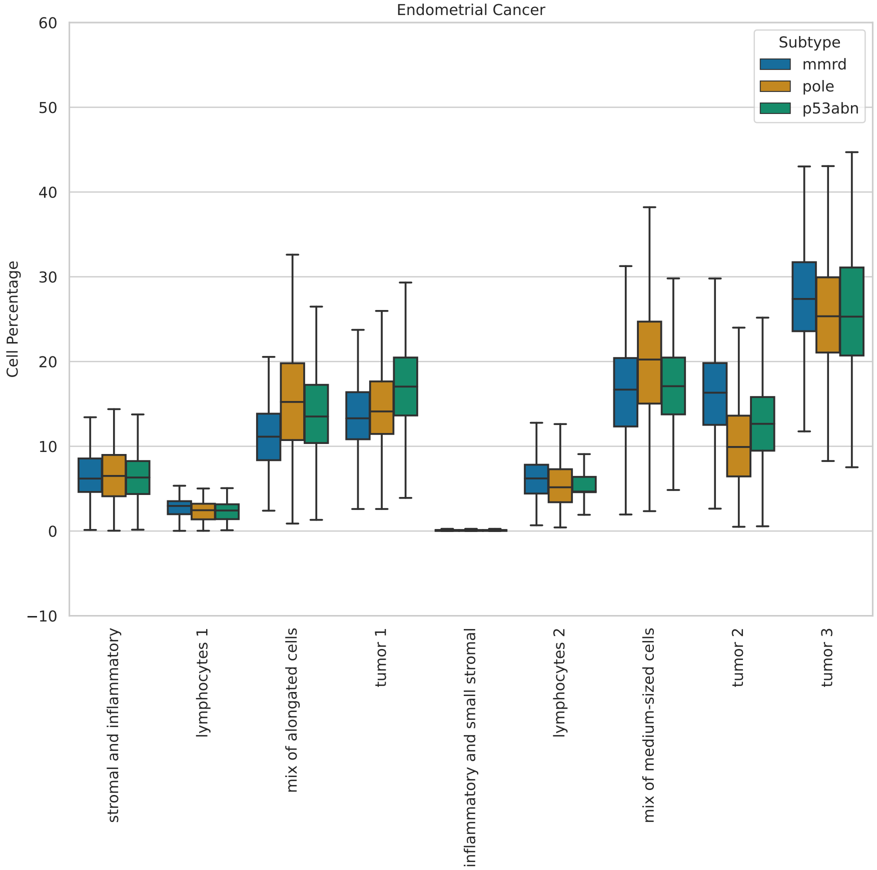

Supplementary Figure 18 Endometrial Cluster Count Distribution. Boxplot of cell cluster distribution across patches of the Endometrial Cancer for all of the patients (Source data are provided as a Source Data file).
