# Supplementary material for: VOLTA: an enVironment-aware cOntrastive ceLl represenTation leArning for histopathology
Source: Nat Commun. 2024 May 10;15:3942. doi: 10.1038/s41467-024-48062-1 (PMC11087497; doi:10.1038/s41467-024-48062-1)
Supplement: Supplementary file 3 — Reporting Summary [file 41467_2024_48062_MOESM3_ESM.pdf]

nature portfolio

Corresponding author(s): Ali Bashashati

Last updated by author(s): May 02, 2024

Reporting Summary

Nature Portfolio wishes to improve the reproducibility of the work that we publish. This form provides structure for consistency and transparency in reporting. For further information on Nature Portfolio policies, see our Editorial Policies and the Editorial Policy Checklist.

Statistics

For all statistical analyses, confirm that the following items are present in the figure legend, table legend, main text, or Methods section.

n/a	Confirmed	
[ ]	[x]	The exact sample size (*n*) for each experimental group/condition, given as a discrete number and unit of measurement
[ ]	[x]	A statement on whether measurements were taken from distinct samples or whether the same sample was measured repeatedly
[x]	[ ]	The statistical test(s) used AND whether they are one- or two-sided<br>*Only common tests should be described solely by name; describe more complex techniques in the Methods section.*
[x]	[ ]	A description of all covariates tested
[x]	[ ]	A description of any assumptions or corrections, such as tests of normality and adjustment for multiple comparisons
[ ]	[x]	A full description of the statistical parameters including central tendency (e.g. means) or other basic estimates (e.g. regression coefficient) AND variation (e.g. standard deviation) or associated estimates of uncertainty (e.g. confidence intervals)
[x]	[ ]	For null hypothesis testing, the test statistic (e.g. *F*, *t*, *r*) with confidence intervals, effect sizes, degrees of freedom and *P* value noted<br>*Give P values as exact values whenever suitable.*
[x]	[ ]	For Bayesian analysis, information on the choice of priors and Markov chain Monte Carlo settings
[x]	[ ]	For hierarchical and complex designs, identification of the appropriate level for tests and full reporting of outcomes
[x]	[ ]	Estimates of effect sizes (e.g. Cohen's *d*, Pearson's *r*), indicating how they were calculated

Our web collection on statistics for biologists contains articles on many of the points above.

Software and code

Policy information about availability of computer code

Data collection	python==3.8.5, pandas==1.3.3, numpy==1.19.5, cikit-image==0.18.3, Pillow==8.3.2, ipython==7.28.0
Data analysis	python==3.8.5, pytorch==1.9.1, torchvision==0.10.1, pandas==1.3.3, numpy==1.19.5, opencv-python==4.5.5.62, scikit-image==0.18.3, scikit-learn==0.24.2, scipy==1.7.1, Pillow==8.3.2, ipython==7.28.0, umap==0.1.1, seaborn==0.11.2, pandas==1.3.3, matplotlib==3.5.1

For manuscripts utilizing custom algorithms or software that are central to the research but not yet described in published literature, software must be made available to editors and reviewers. We strongly encourage code deposition in a community repository (e.g. GitHub). See the Nature Portfolio guidelines for submitting code & software for further information.

Data

Policy information about availability of data

All manuscripts must include a data availability statement. This statement should provide the following information, where applicable:

- Accession codes, unique identifiers, or web links for publicly available datasets
- A description of any restrictions on data availability
- For clinical datasets or third party data, please ensure that the statement adheres to our policy

CoNSeP https://warwick.ac.uk/fac/cross_fac/tia/data/hovernet/
NuCLS: https://sites.google.com/view/nucls/home

PanNuke: https://arxiv.org/abs/2003.10778
Lizard: https://arxiv.org/abs/2108.11195, MiDOG: https://arxiv.org/pdf/2204.03742
All the internal histopathology slides are available through direct email to the corresponding author. Access will be provided after institutional & ethical permissions.

Research involving human participants, their data, or biological material

Policy information about studies with human participants or human data. See also policy information about sex, gender (identity/presentation), and sexual orientation and race, ethnicity and racism.

Reporting on sex and gender	Our study does not have any restrictions on sex and gender of the subjects. To the best of our knowledge, the public datasets used in this study do not apply any restrictions in this context as well. However, the breast and ovarian datasets only include female subjects.
Reporting on race, ethnicity, or other socially relevant groupings	Our study does not have any restrictions on race, ethnicity, or socially relevant groupings of the subjects. Additionally, these types of information are not available for the public datasets used in this study.
Population characteristics	Our study includes experiments on seven datasets collected from different geographical locations. However, to our knowledge, the demographic information of the patients (e.g., age, current treatment) is not available for the public datasets used in this study. However, we believe this type of information does not directly impact the nature of our task, which is representing the characteristics of the cells within the tissue.
Recruitment	To our knowledge, this information is not available for the public datasets used in this study. However, we have included seven datasets from different clinical centers to mitigate any potential biases.
Ethics oversight	n/a

Note that full information on the approval of the study protocol must also be provided in the manuscript.

Field-specific reporting

Please select the one below that is the best fit for your research. If you are not sure, read the appropriate sections before making your selection.

[x] Life sciences   [ ] Behavioural & social sciences   [ ] Ecological, evolutionary & environmental sciences

For a reference copy of the document with all sections, see nature.com/documents/nr-reporting-summary-flat.pdf

Life sciences study design

All studies must disclose on these points even when the disclosure is negative.

Sample size	The sample size is chosen based on the setting provided in the previous studies that introduced each of the datasets. These sample sizes include more than 17,000 samples in each dataset, which is more than enough for training and testing a deep-learning model. Additionally, our extensive experiments on large dataset including 280,000 cell samples show the robustness and validity of the results across scale.
Data exclusions	We did not excluded any data.
Replication	We ran each experiment multiple times and measured the average of the results due to the stochastic nature of the training process. Even though the exact replication is not possible due to the stochastic nature, the final results of different experiments have minor differences. Additionally, the pre-trained weights are available which can be used for reproduction of the results.
Randomization	Where available, the splitting of the data was taken from the setting that the original publication of each dataset suggested. In the rest of the scenarios, we used a randomized patient-wise selection process.
Blinding	The used datasets in this study are public, and to the best of our knowledge, the data collection process was blind. Our approach was a blind process as well. We used the patient stratification from that of the original publications for the public datasets. For the private datasets, we used random stratification in a blind manner.

Reporting for specific materials, systems and methods

We require information from authors about some types of materials, experimental systems and methods used in many studies. Here, indicate whether each material, system or method listed is relevant to your study. If you are not sure if a list item applies to your research, read the appropriate section before selecting a response.

Materials & experimental systems

n/a	Involved in the study
☒	☐ Antibodies
☒	☐ Eukaryotic cell lines
☒	☐ Palaeontology and archaeology
☒	☐ Animals and other organisms
☒	☐ Clinical data
☒	☐ Dual use research of concern
☒	☐ Plants

Methods

n/a	Involved in the study
☒	☐ ChIP-seq
☒	☐ Flow cytometry
☒	☐ MRI-based neuroimaging

Plants

Seed stocks	n/a
Novel plant genotypes	n/a
Authentication	n/a
