# Supplementary material for: VOLTA: an enVironment-aware cOntrastive ceLl represenTation leArning for histopathology
Source: Nat Commun. 2024 May 10;15:3942. doi: 10.1038/s41467-024-48062-1 (PMC11087497; doi:10.1038/s41467-024-48062-1)
Supplement: Supplementary file 4 — Source Data [file 41467_2024_48062_MOESM4_ESM.zip › source data/figures/Supplementary Figure 7.pptx]

## Slide 1
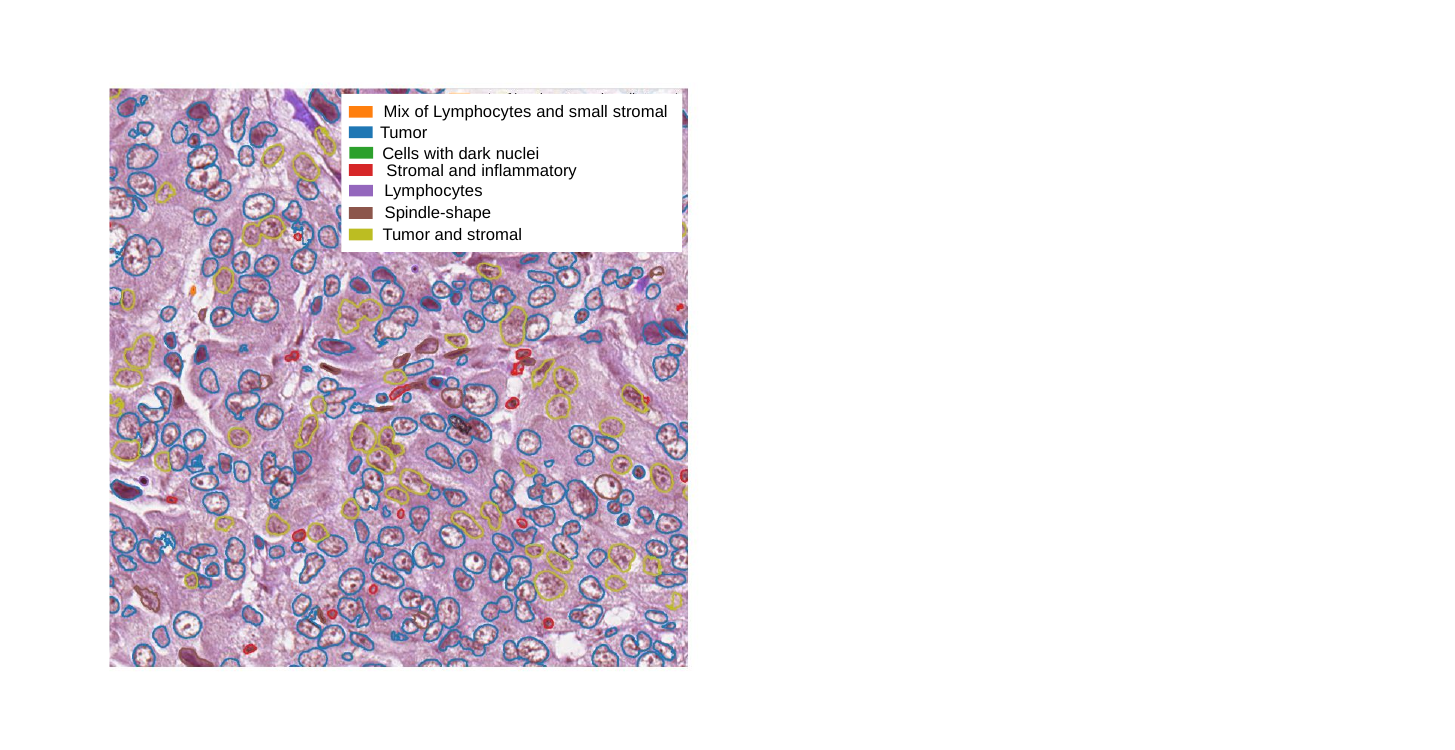

Mix of Lymphocytes and small stromal
Tumor
Cells with dark nuclei
Stromal and inflammatory
Lymphocytes
Spindle-shape
Tumor and stromal
