# Supplementary figures and images for: VOLTA: an enVironment-aware cOntrastive ceLl represenTation leArning for histopathology
Source: Nat Commun. 2024 May 10;15:3942. doi: 10.1038/s41467-024-48062-1 (PMC11087497; doi:10.1038/s41467-024-48062-1)

Endometrial Cancer

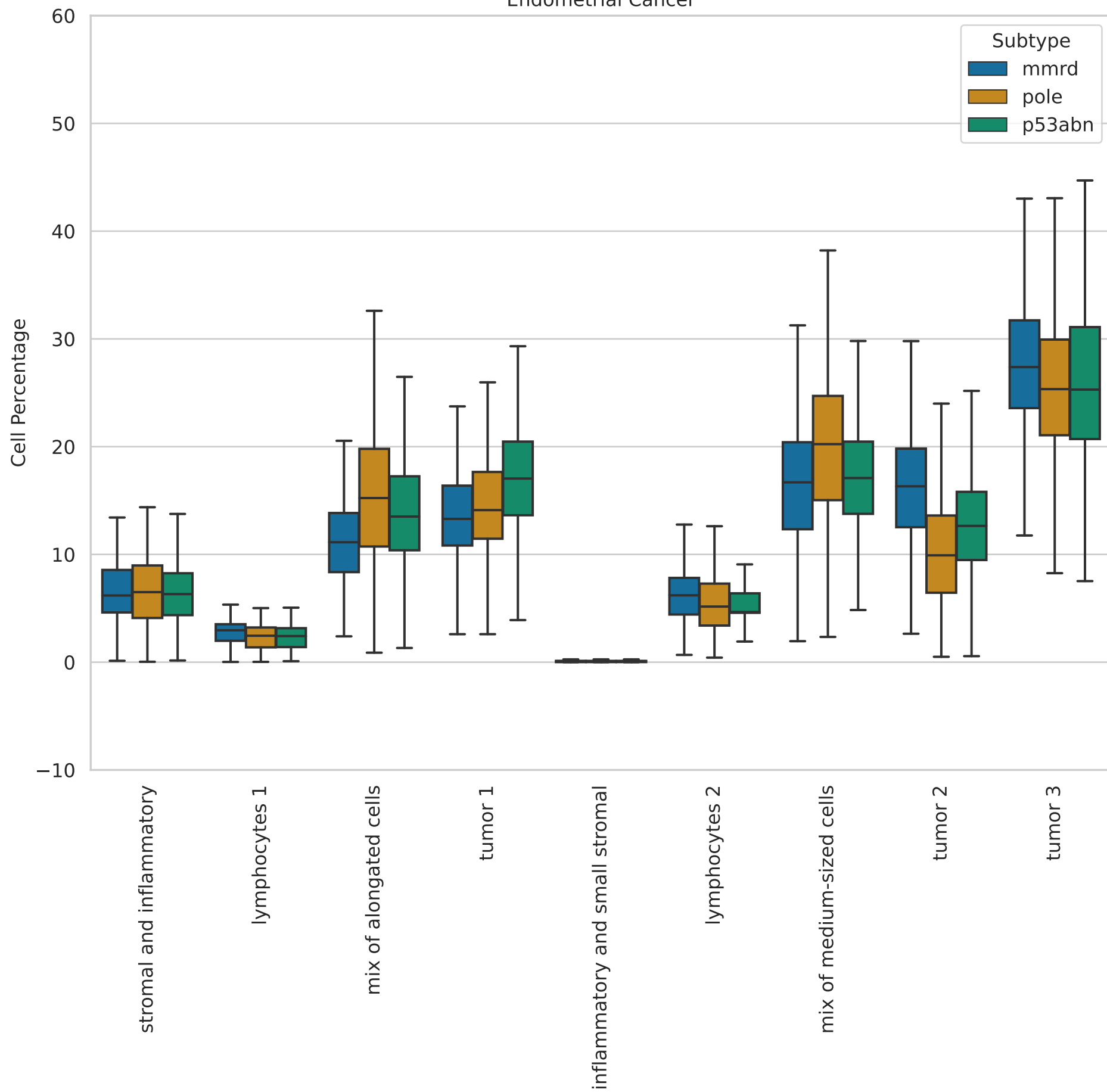

Supplement: Supplementary file 4 — Source Data [file 41467_2024_48062_MOESM4_ESM.zip › source data/figures/Supplementary Figure 18.pdf]

(a) CoNSeP

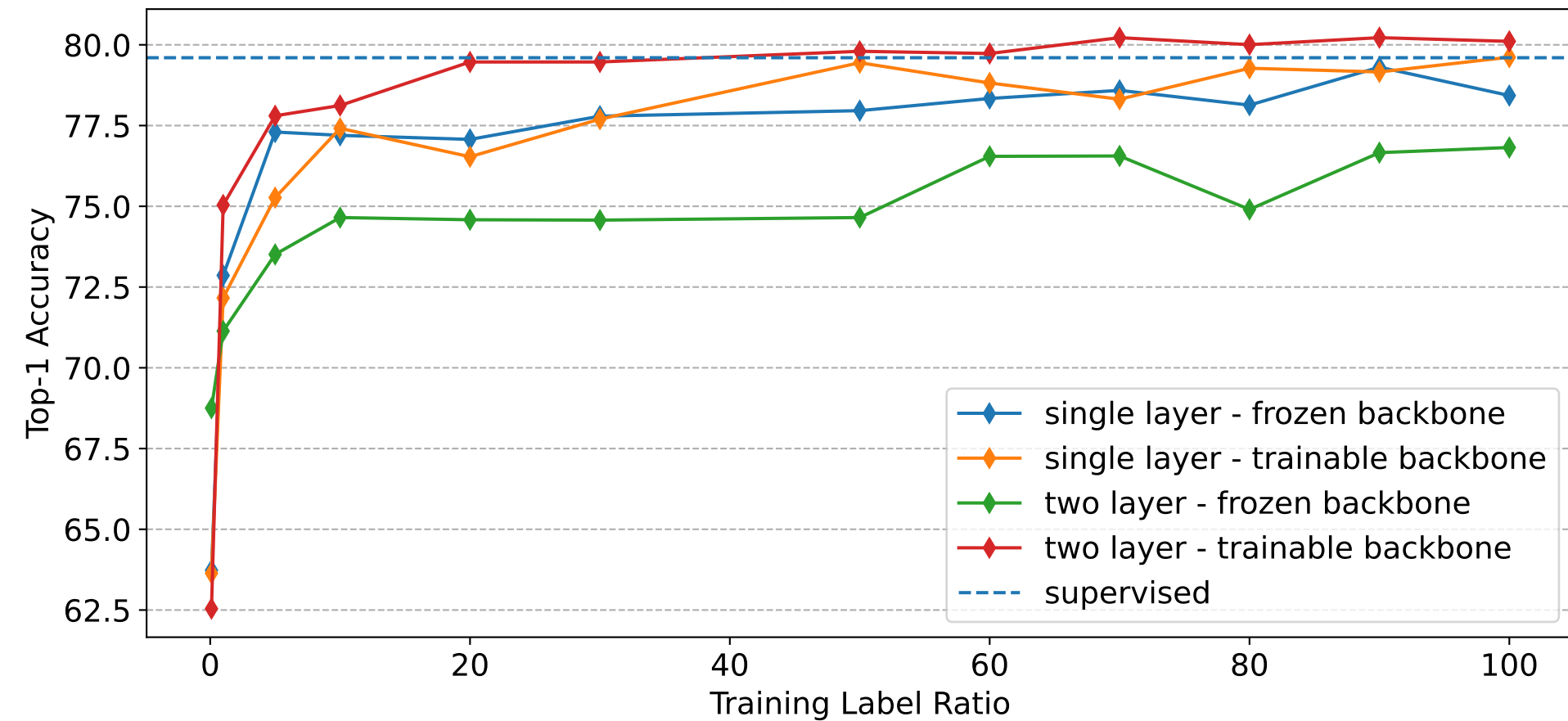

(b) NuCLS

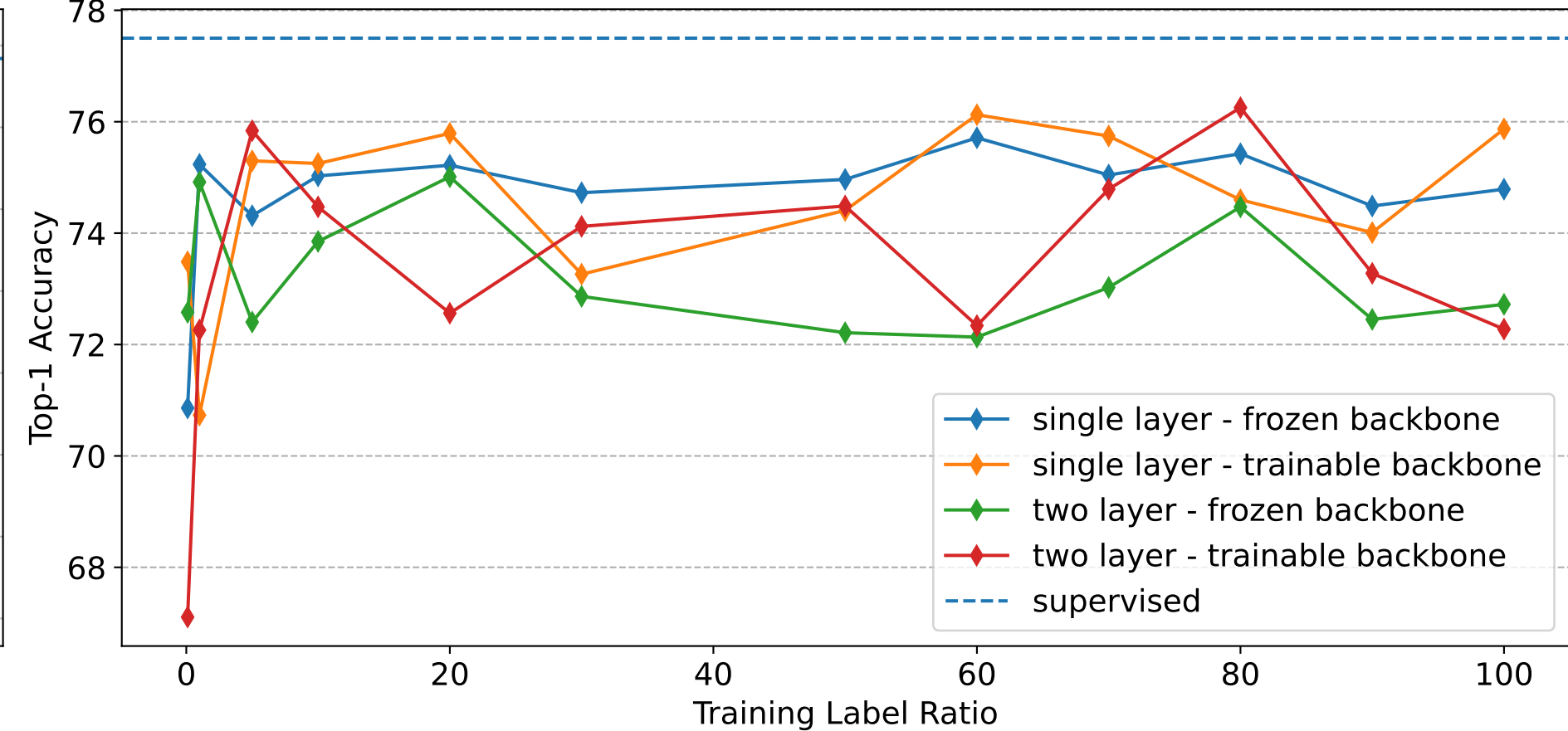

(c) NuCLS with Stain Normalization

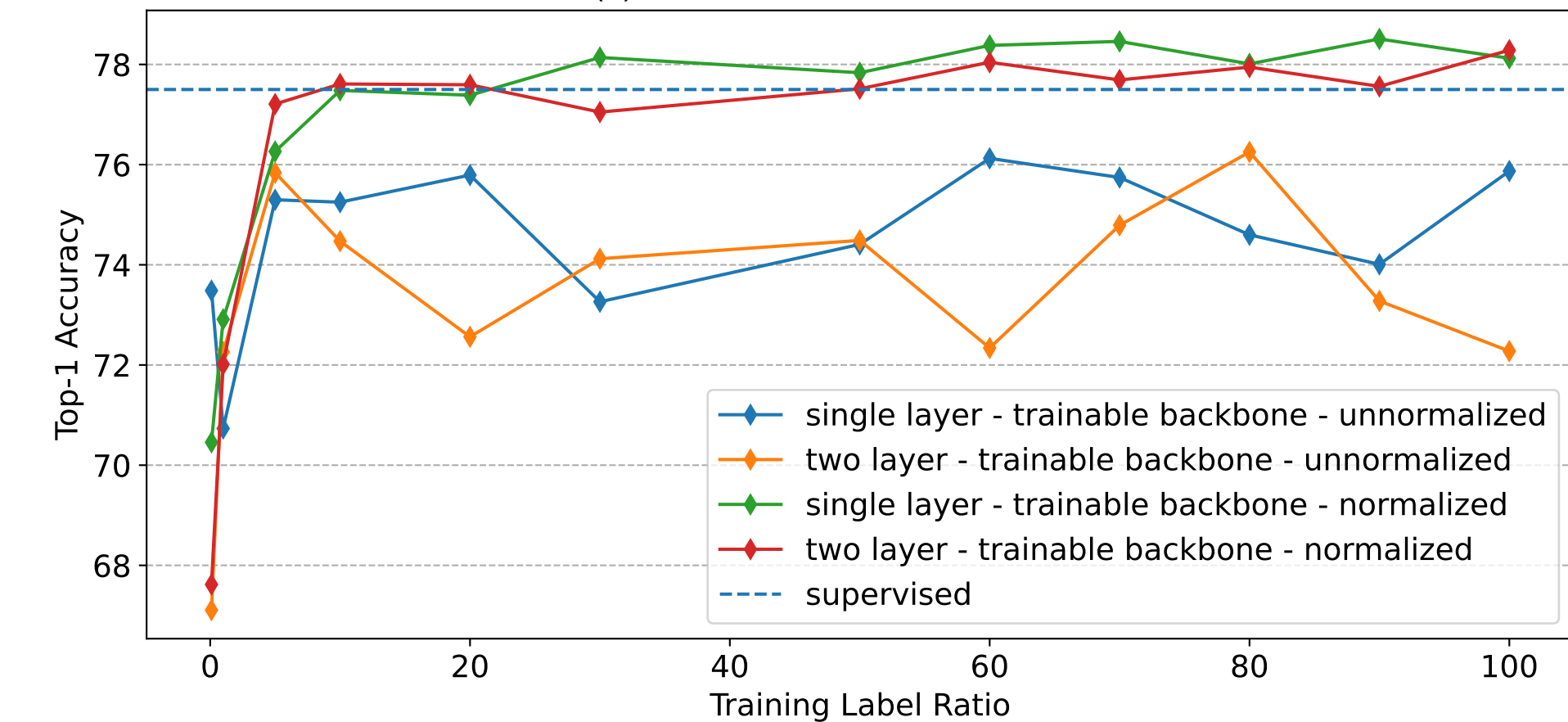

Supplement: Supplementary file 4 — Source Data [file 41467_2024_48062_MOESM4_ESM.zip › source data/figures/Figure 3.pdf]

CoNsep

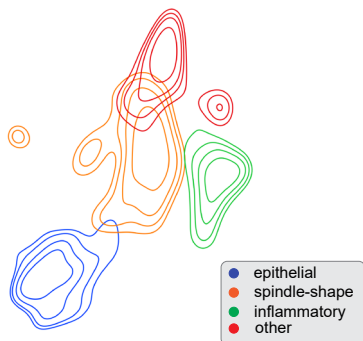

NuCLS

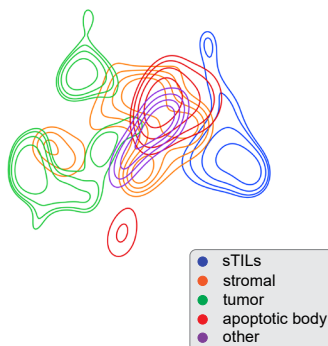

PanNuke Colon

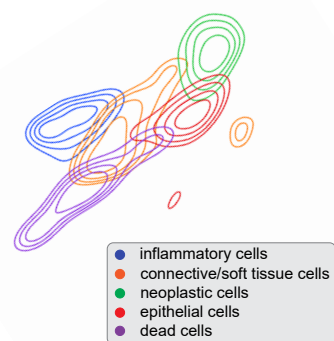

PanNuke Breast

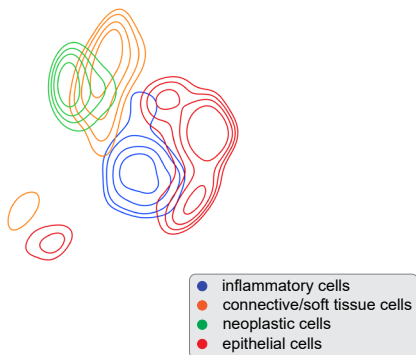

lizard

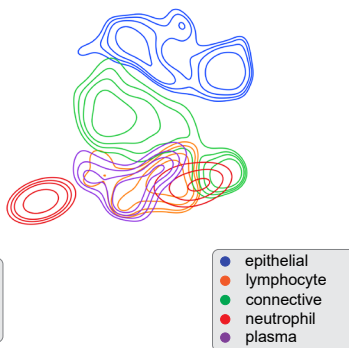

Oracle

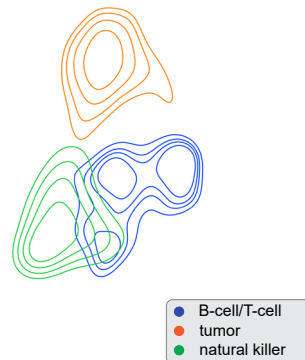

SarcCell

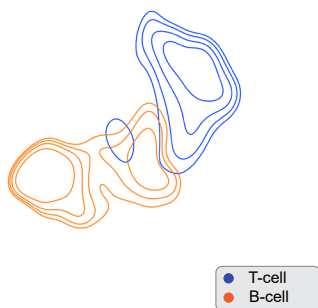

Mast Cell

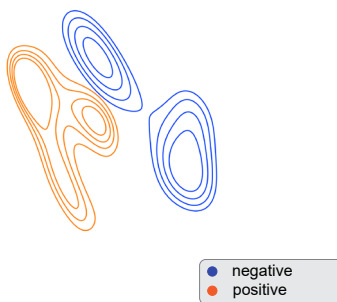

Midog

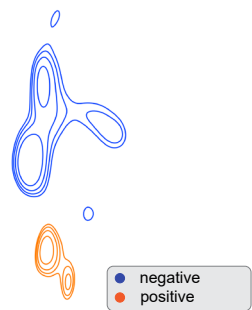

Supplement: Supplementary file 4 — Source Data [file 41467_2024_48062_MOESM4_ESM.zip › source data/figures/Figure 2.pdf]

## Environment Block

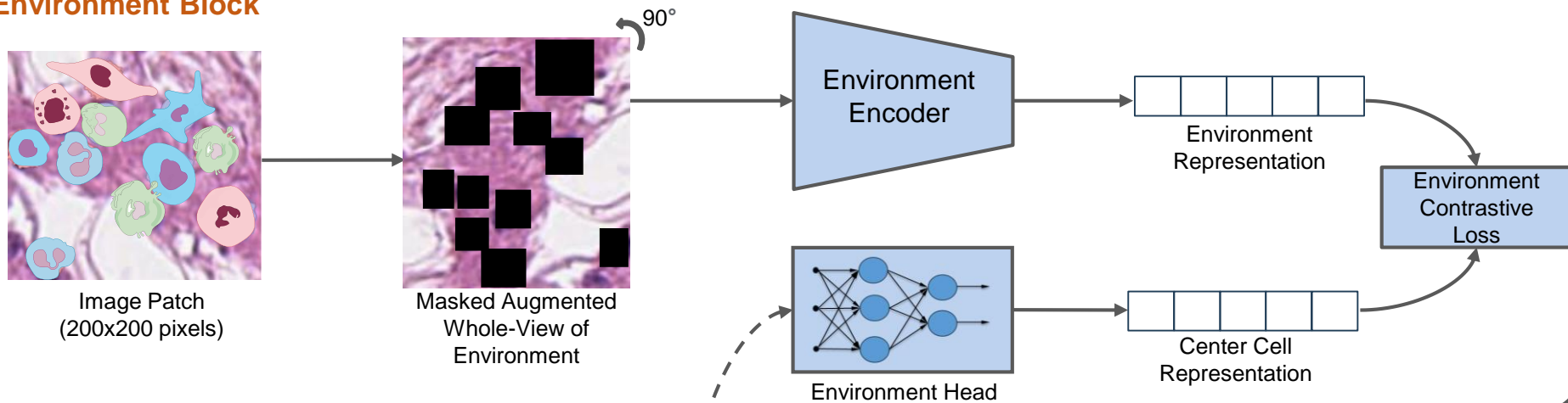

## Cell Block

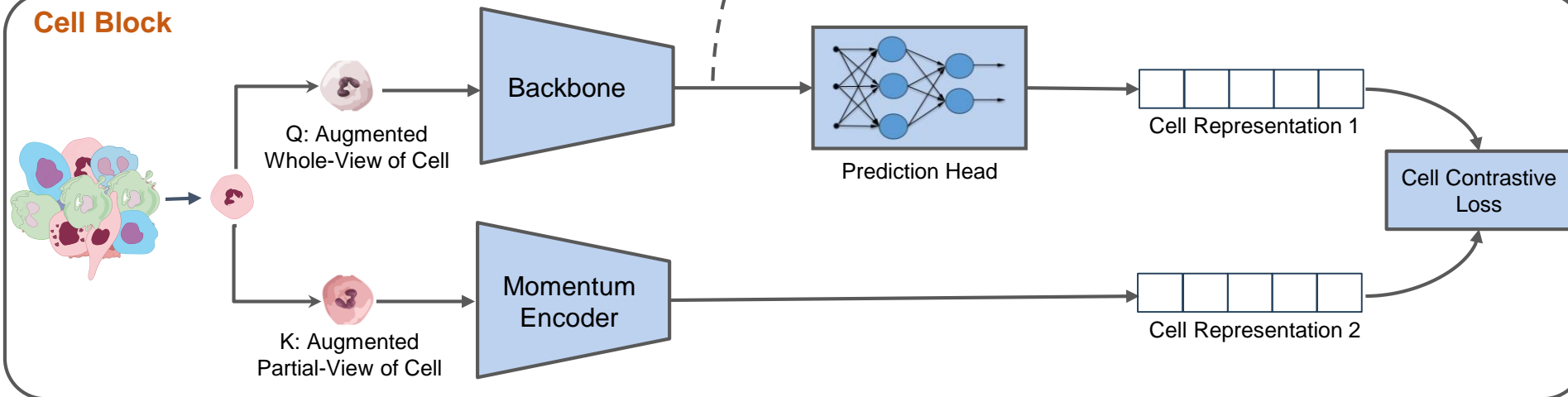

Supplement: Supplementary file 4 — Source Data [file 41467_2024_48062_MOESM4_ESM.zip › source data/figures/Figure 1.pdf]

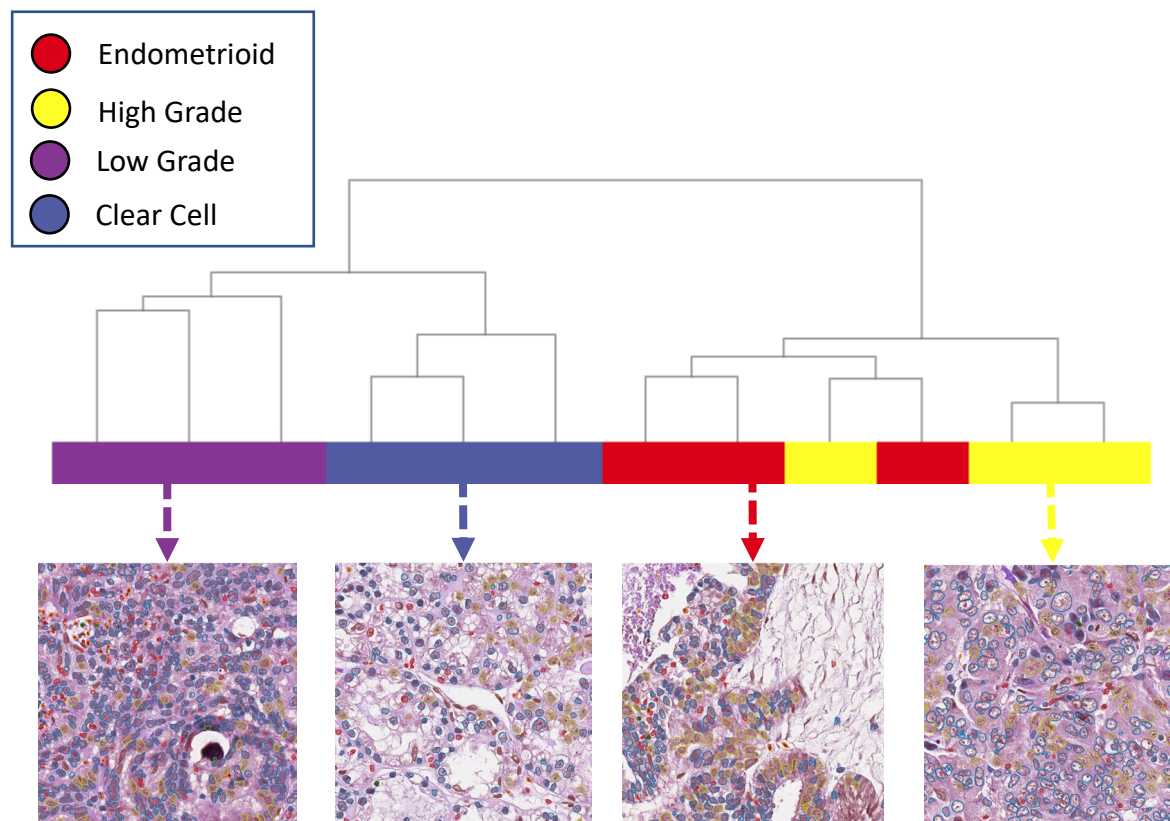

(a) Ovarian Dataset 1

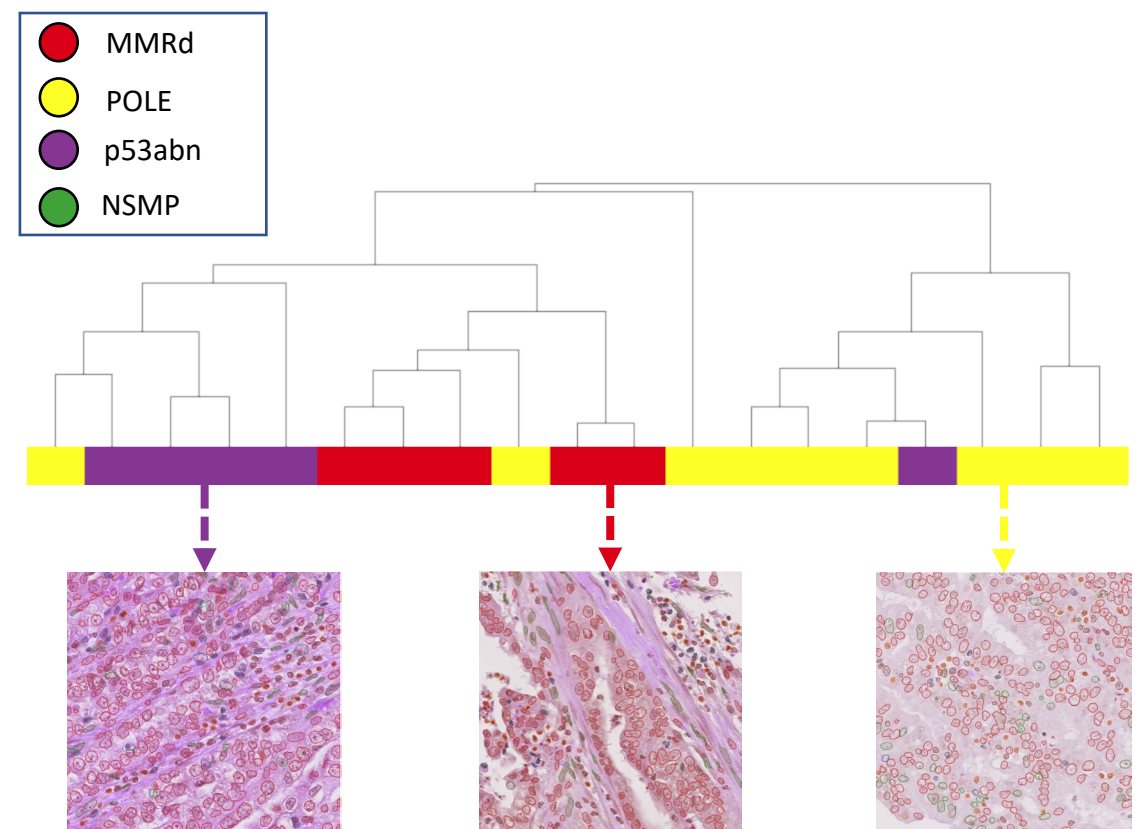

(b) Endometrial Dataset 1

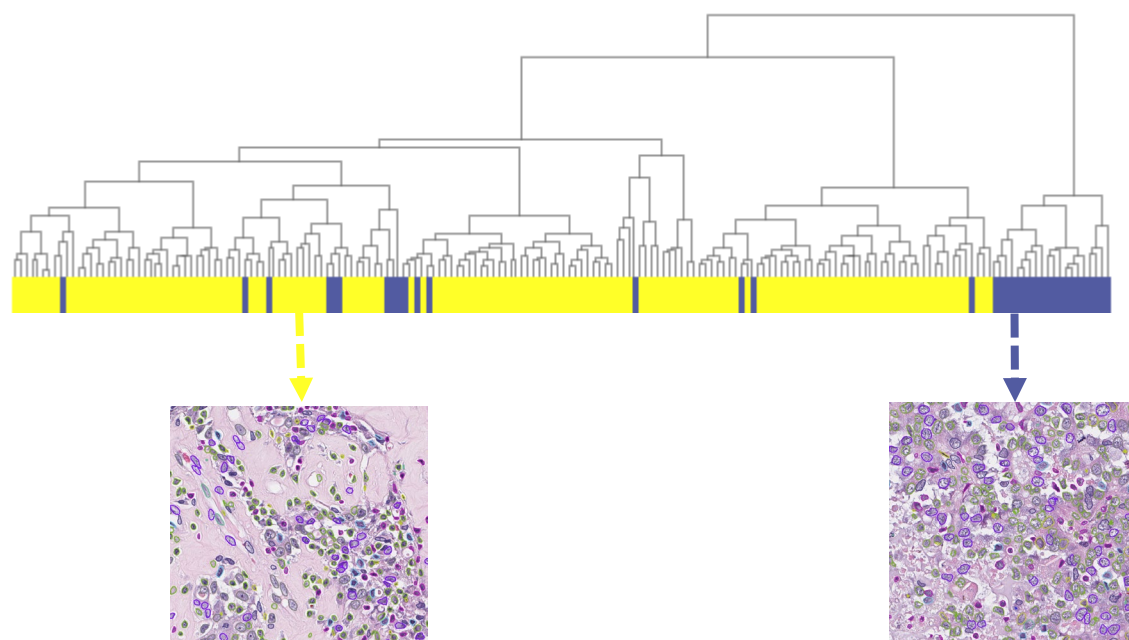

(c) Ovarian Dataset 2

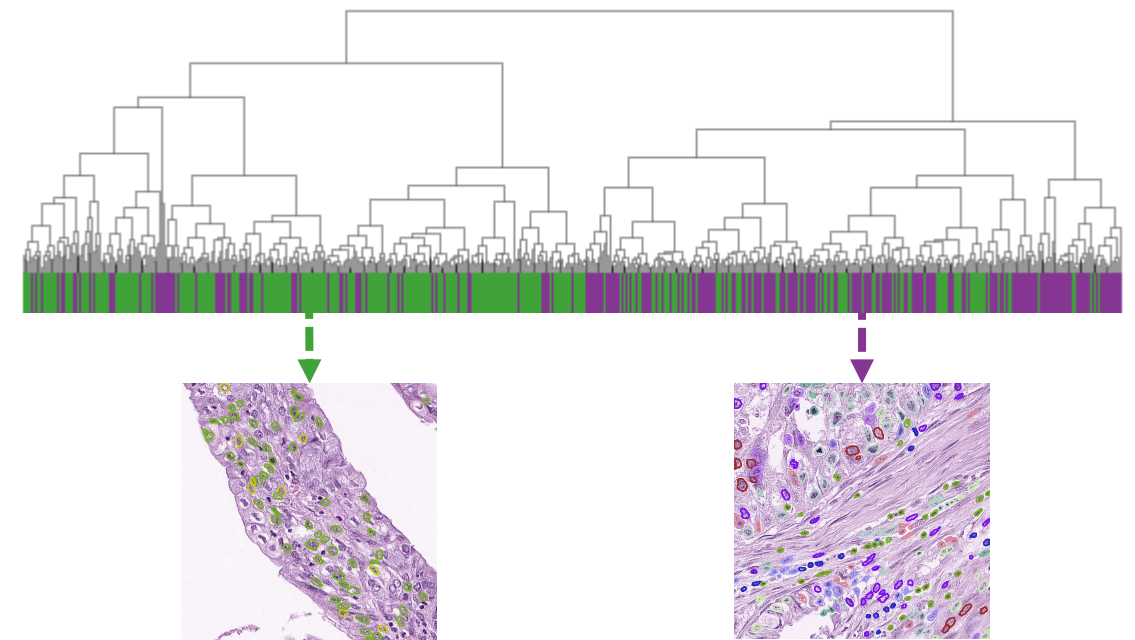

(d) Endometrial Dataset 2

Supplement: Supplementary file 4 — Source Data [file 41467_2024_48062_MOESM4_ESM.zip › source data/figures/Figure 4.pdf]

CoNSep

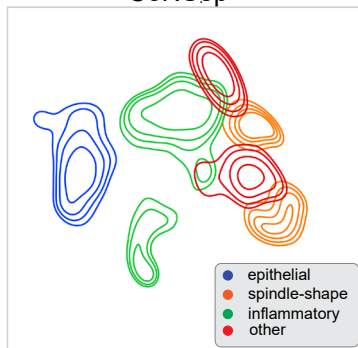

NuCLS

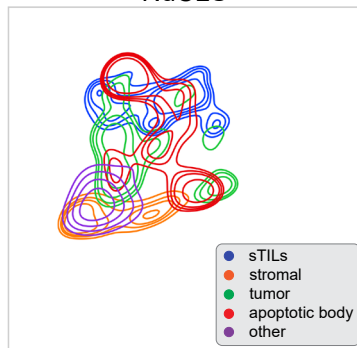

PanNuke Colon

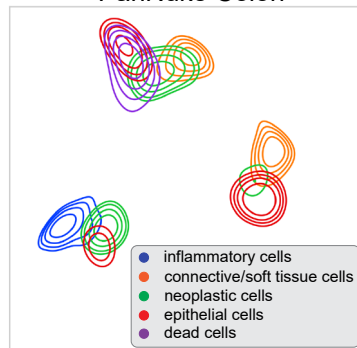

PanNuke Breast

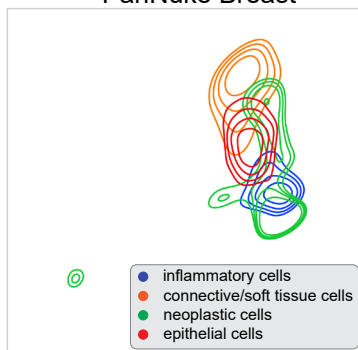

lizard

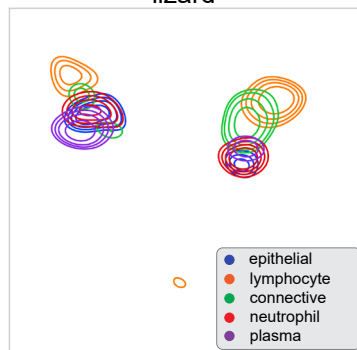

Oracle

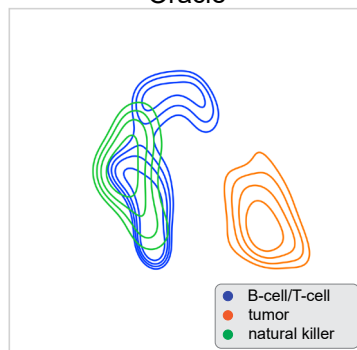

SarcCell

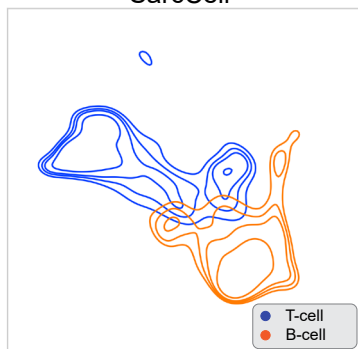

Mast Cell

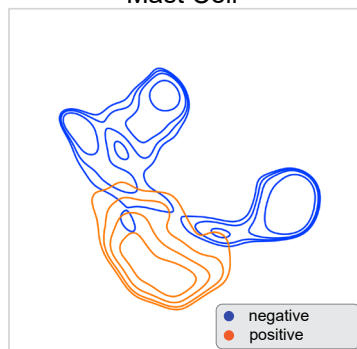

Midog

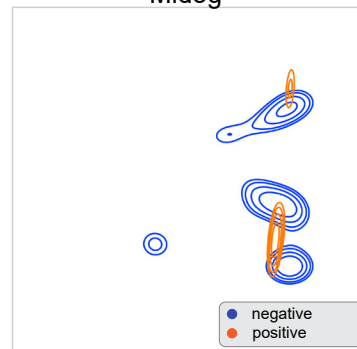

Supplement: Supplementary file 4 — Source Data [file 41467_2024_48062_MOESM4_ESM.zip › source data/figures/Supplementary Figure 4.pdf]

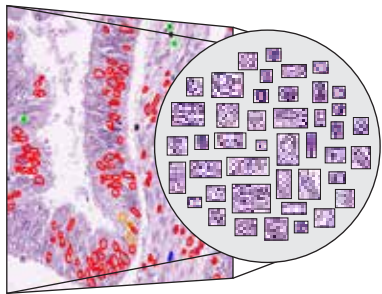

Cell Extraction

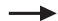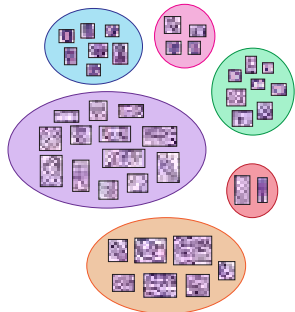

Cell Clustering

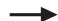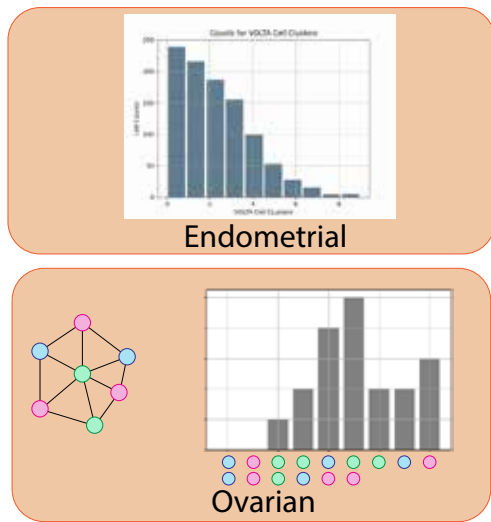

Statistical Representation

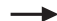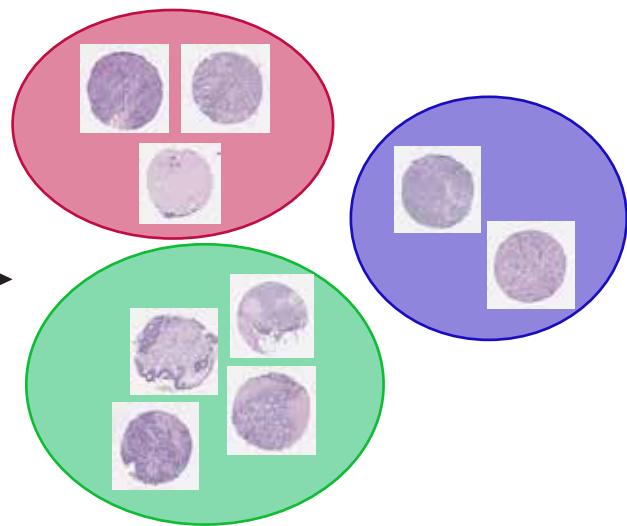

Cancer Subtyping

Supplement: Supplementary file 4 — Source Data [file 41467_2024_48062_MOESM4_ESM.zip › source data/figures/Supplementary Figure 5.pdf]

CoNSep

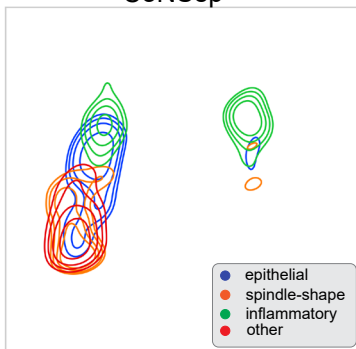

NuCLS

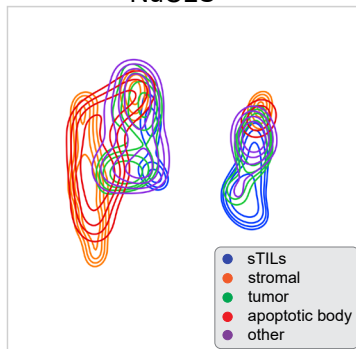

PanNuke Colon

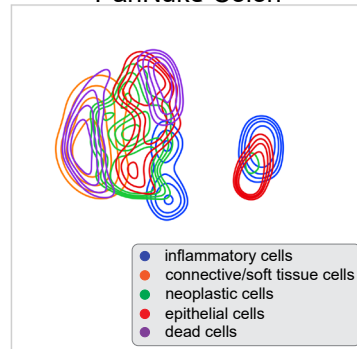

PanNuke Breast

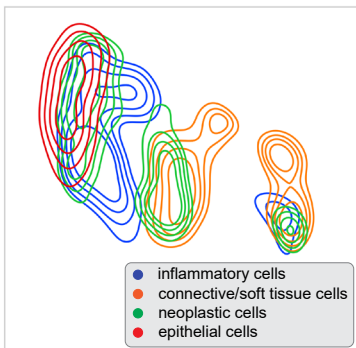

lizard

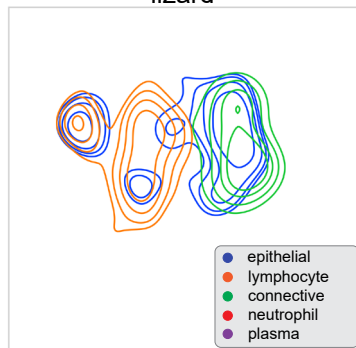

SarcCell

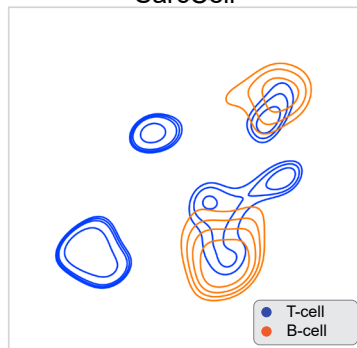

Mast Cell

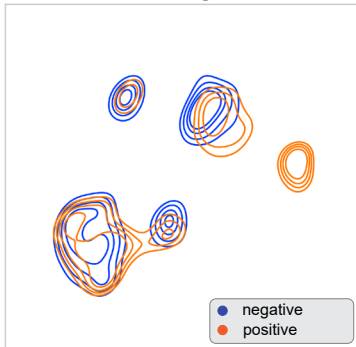

Midog

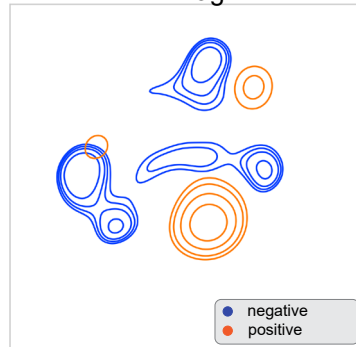

Supplement: Supplementary file 4 — Source Data [file 41467_2024_48062_MOESM4_ESM.zip › source data/figures/Supplementary Figure 2.pdf]

CoNSep

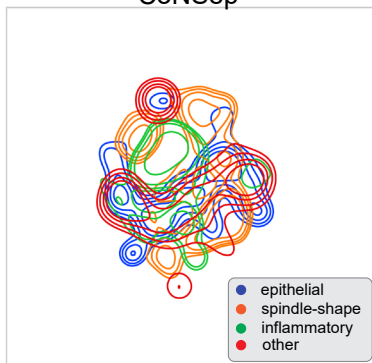

NuCLS

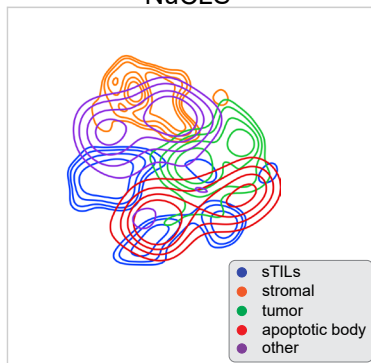

PanNuke Colon

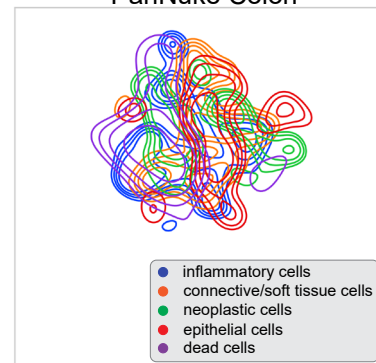

PanNuke Breast

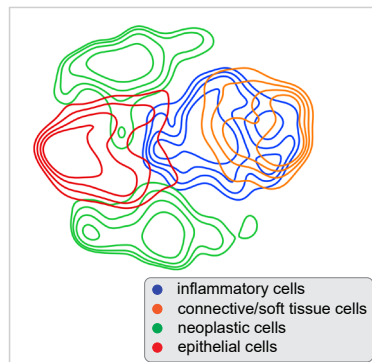

lizard

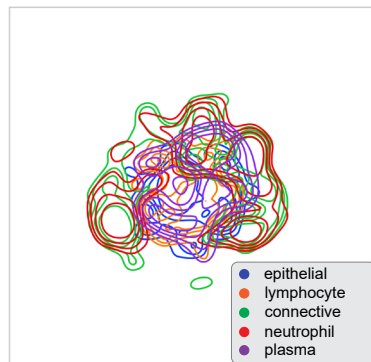

Supplement: Supplementary file 4 — Source Data [file 41467_2024_48062_MOESM4_ESM.zip › source data/figures/Supplementary Figure 3.pdf]

CoNSep

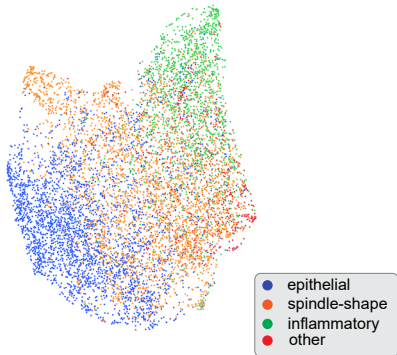

NuCLS

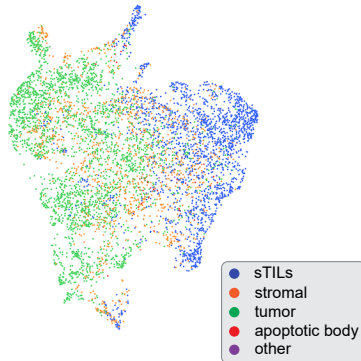

PanNuke Colon

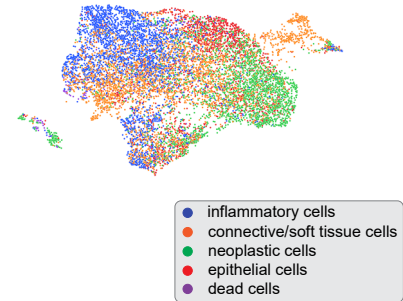

PanNuke Breast

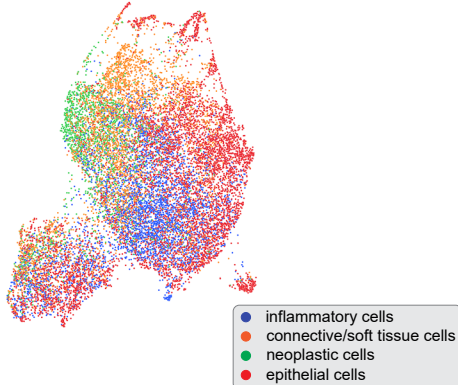

lizard

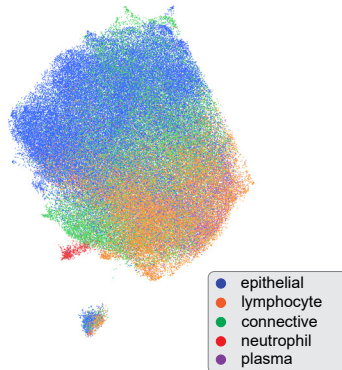

Oracle

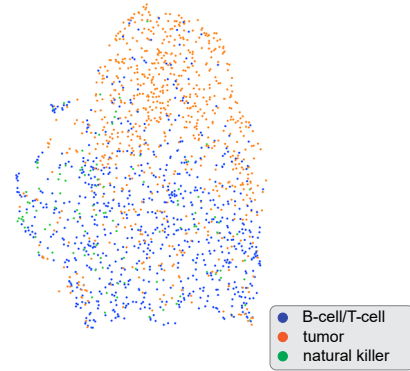

SarcCell

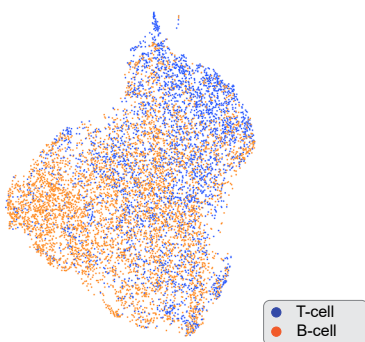

Mast Cell

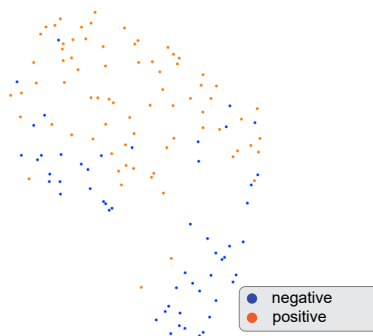

Midog

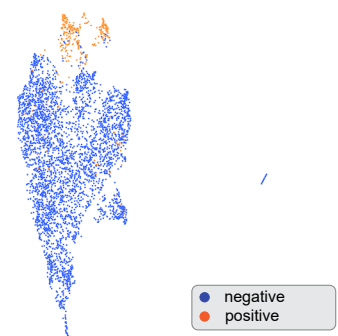

Supplement: Supplementary file 4 — Source Data [file 41467_2024_48062_MOESM4_ESM.zip › source data/figures/Supplementary Figure 1.pdf]

Ovarian Cancer

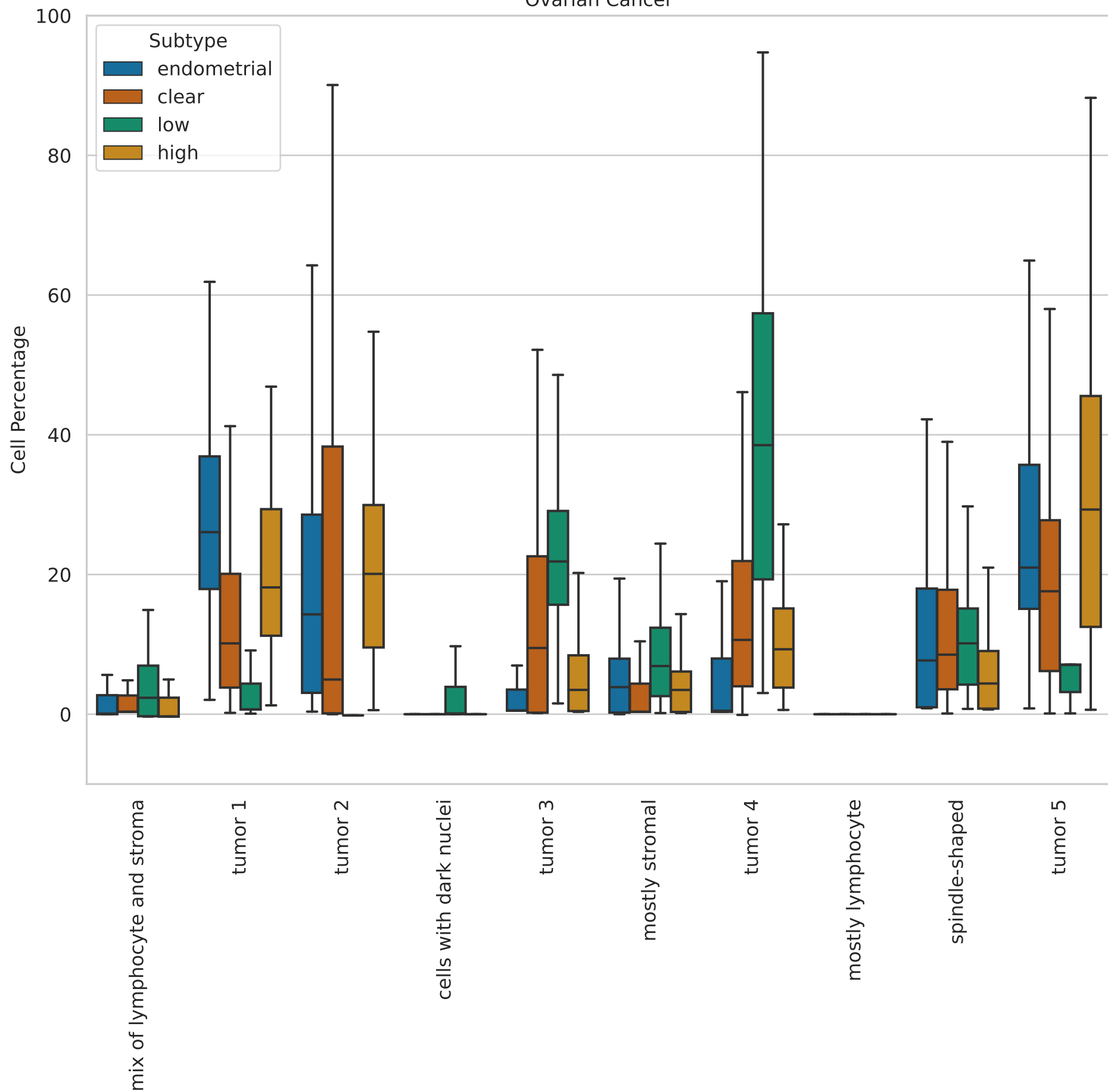

Supplement: Supplementary file 4 — Source Data [file 41467_2024_48062_MOESM4_ESM.zip › source data/figures/Supplementary Figure 11.pdf]

Tumor 2, 4, and 5 ratio other tumor groups

## Ovarian Cancer

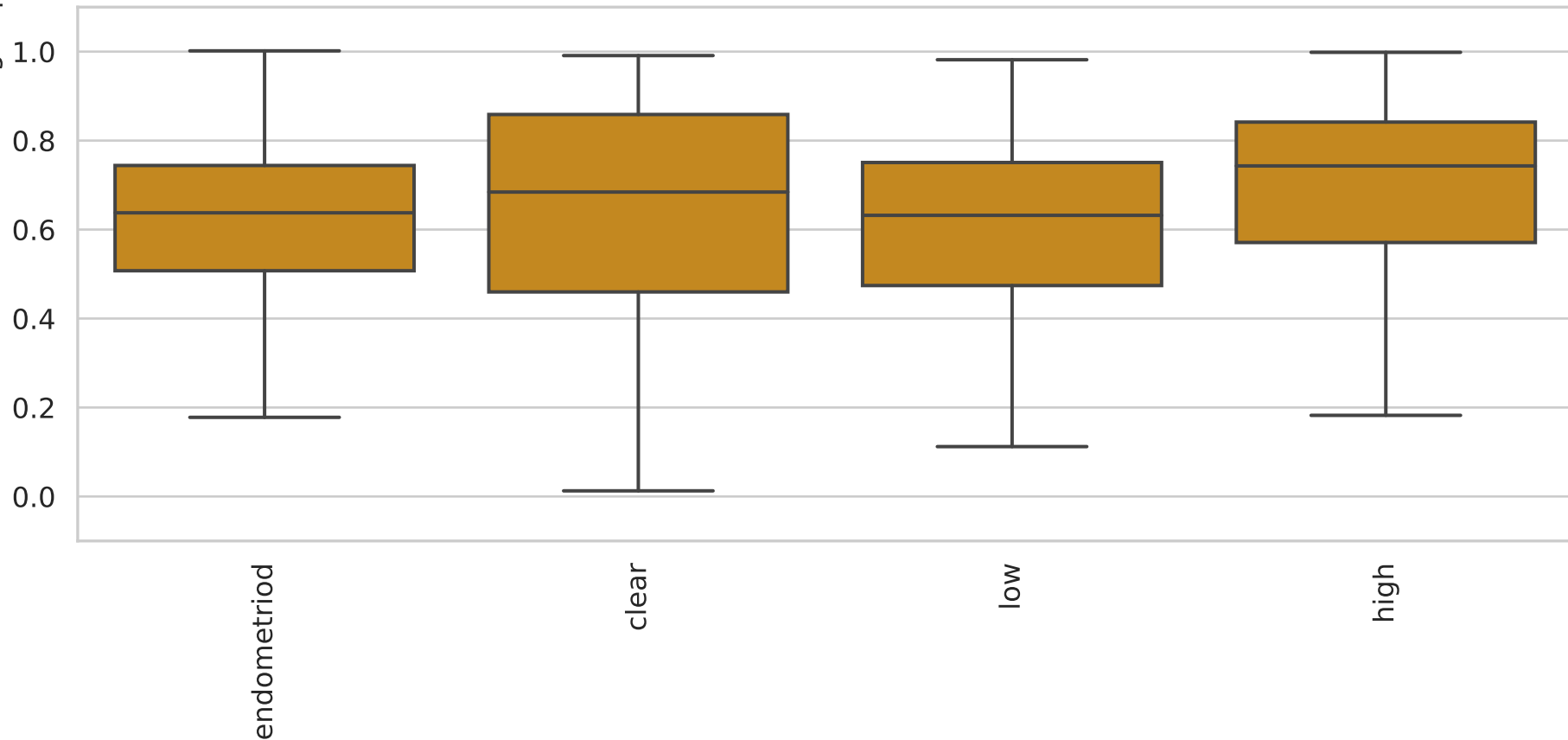

Supplement: Supplementary file 4 — Source Data [file 41467_2024_48062_MOESM4_ESM.zip › source data/figures/Supplementary Figure 13.pdf]

## Ovarian Cancer

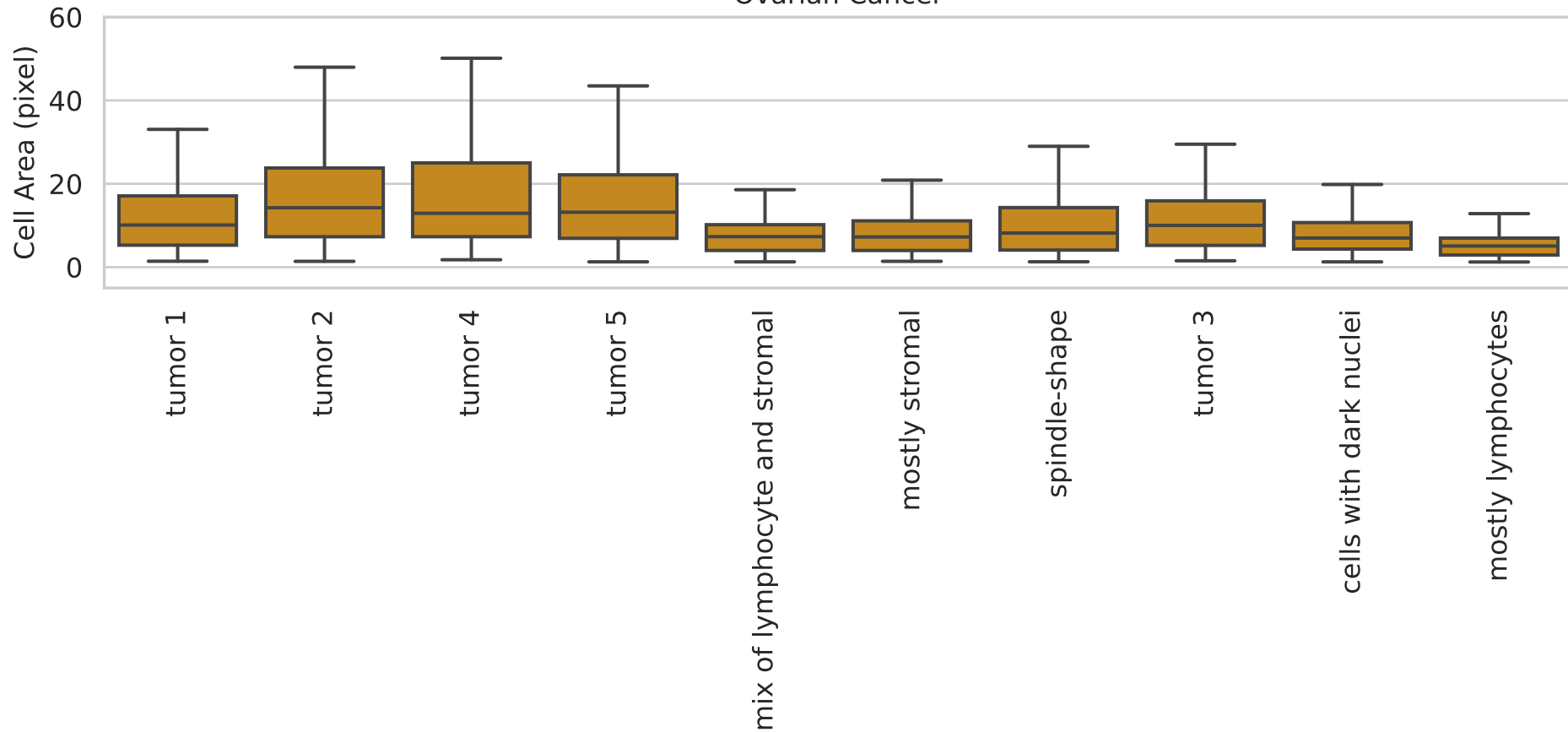

Supplement: Supplementary file 4 — Source Data [file 41467_2024_48062_MOESM4_ESM.zip › source data/figures/Supplementary Figure 12.pdf]
